# A 0/1h-algorithm using cardiac myosin-binding protein C for early diagnosis of myocardial infarction

**DOI:** 10.1093/ehjacc/zuac007

**Published:** 2022-02-12

**Authors:** Thomas E Kaier, Raphael Twerenbold, Pedro Lopez-Ayala, Thomas Nestelberger, Jasper Boeddinghaus, Bashir Alaour, Iris-Martina Huber, Yuan Zhi, Luca Koechlin, Desiree Wussler, Karin Wildi, Samyut Shrestha, Ivo Strebel, Oscar Miro, Javier F Martín-Sánchez, Michael Christ, Damien Kawecki, Dagmar I Keller, Maria Rubini Gimenez, Michael Marber, Christian Mueller, APACE Investigators, Michael Freese, Paul David Ratmann, Alexandra Prepoudis, Danielle M Gualandro, Nicolas Geigy, Tobias Reichlin, Katharina Rentsch, Mario Maier, Valentina Troester, Juliane Gehrke, Tania Coscia, Noemi Glarner, Hadrien Schoepfer, Michael Buechi, Joan Walter, Ana Yufera Sanchez, Christian Puelacher, Jeanne du Fay de Lavallaz, Alessandra Sanzione, Ibrahim Schäfer, Petra Hillinger, Beatriz López, Esther Rodriguez Adrada, Piotr Muzyk, Beata Morawiec, Jiri Parenica, Eva Ganovská, Jens Lohrmann, Andreas Buser, Arnold von Eckardstein, Roland Bingisser, Christian Nickel

**Affiliations:** King’s College London BHF Centre, The Rayne Institute, St Thomas’ Hospital, London, UK; Department of Cardiology, Cardiovascular Research Institute Basel (CRIB), University Hospital Basel, University of Basel, Petersgraben 4, CH-4031 Basel, Switzerland; GREAT Network; University Center of Cardiovascular Science and Department of Cardiology, University Heart and Vascular Center Hamburg, Hamburg, Germany; Department of Cardiology, Cardiovascular Research Institute Basel (CRIB), University Hospital Basel, University of Basel, Petersgraben 4, CH-4031 Basel, Switzerland; GREAT Network; Department of Cardiology, Cardiovascular Research Institute Basel (CRIB), University Hospital Basel, University of Basel, Petersgraben 4, CH-4031 Basel, Switzerland; GREAT Network; Division of Cardiology, Vancouver General Hospital, University of British Columbia, Vancouver, British Columbia, Canada; Department of Cardiology, Cardiovascular Research Institute Basel (CRIB), University Hospital Basel, University of Basel, Petersgraben 4, CH-4031 Basel, Switzerland; GREAT Network; King’s College London BHF Centre, The Rayne Institute, St Thomas’ Hospital, London, UK; Department of Cardiology, Cardiovascular Research Institute Basel (CRIB), University Hospital Basel, University of Basel, Petersgraben 4, CH-4031 Basel, Switzerland; GREAT Network; Department of Cardiology, Cardiovascular Research Institute Basel (CRIB), University Hospital Basel, University of Basel, Petersgraben 4, CH-4031 Basel, Switzerland; GREAT Network; Department of Cardiology, Cardiovascular Research Institute Basel (CRIB), University Hospital Basel, University of Basel, Petersgraben 4, CH-4031 Basel, Switzerland; GREAT Network; Department of Cardiac Surgery, University Hospital Basel, University of Basel, Basel, Switzerland; Department of Cardiology, Cardiovascular Research Institute Basel (CRIB), University Hospital Basel, University of Basel, Petersgraben 4, CH-4031 Basel, Switzerland; GREAT Network; Department of Cardiology, Cardiovascular Research Institute Basel (CRIB), University Hospital Basel, University of Basel, Petersgraben 4, CH-4031 Basel, Switzerland; GREAT Network; Critical Care Research Institute, The Prince Charles Hospital, Brisbane, Queensland, Australia; University of Queensland, Brisbane, Queensland, Australia; Department of Cardiology, Cardiovascular Research Institute Basel (CRIB), University Hospital Basel, University of Basel, Petersgraben 4, CH-4031 Basel, Switzerland; GREAT Network; Department of Cardiology, Cardiovascular Research Institute Basel (CRIB), University Hospital Basel, University of Basel, Petersgraben 4, CH-4031 Basel, Switzerland; GREAT Network; Department of Cardiology, Cardiovascular Research Institute Basel (CRIB), University Hospital Basel, University of Basel, Petersgraben 4, CH-4031 Basel, Switzerland; GREAT Network; Emergency Department, Hospital Clinic, Barcelona, Catalonia, Spain; Department of Cardiology, Cardiovascular Research Institute Basel (CRIB), University Hospital Basel, University of Basel, Petersgraben 4, CH-4031 Basel, Switzerland; GREAT Network; Emergency Department, Hospital Clinico San Carlos, Madrid, Spain; GREAT Network; Department of Emergency Medicine, Luzerner Kantonsspital, Lucerne, Switzerland; Department of Cardiology, Cardiovascular Research Institute Basel (CRIB), University Hospital Basel, University of Basel, Petersgraben 4, CH-4031 Basel, Switzerland; GREAT Network; Emergency Department, University Hospital Zurich, Zurich, Switzerland; Department of Cardiology, Cardiovascular Research Institute Basel (CRIB), University Hospital Basel, University of Basel, Petersgraben 4, CH-4031 Basel, Switzerland; GREAT Network; Department of Internal Medicine/Cardiology, Heart Center Leipzig, University of Leipzig, Leipzig Heart Institute, 04289 Leipzig, Germany; King’s College London BHF Centre, The Rayne Institute, St Thomas’ Hospital, London, UK; Department of Cardiology, Cardiovascular Research Institute Basel (CRIB), University Hospital Basel, University of Basel, Petersgraben 4, CH-4031 Basel, Switzerland; GREAT Network

**Keywords:** Cardiac myosin-binding protein C, cMyC, Troponin I, Troponin T, Myocardial infarction, APACE

## Abstract

**Aims:**

Cardiac myosin-binding protein C (cMyC) demonstrated high diagnostic accuracy for the early detection of non-ST-elevation myocardial infarction (NSTEMI). Its dynamic release kinetics may enable a 0/1h-decision algorithm that is even more effective than the ESC hs-cTnT/I 0/1 h rule-in/rule-out algorithm.

**Methods and results:**

In a prospective international diagnostic study enrolling patients presenting with suspected NSTEMI to the emergency department, cMyC was measured at presentation and after 1 h in a blinded fashion. Modelled on the ESC hs-cTnT/I 0/1h-algorithms, we derived a 0/1h-cMyC-algorithm. Final diagnosis of NSTEMI was centrally adjudicated according to the 4th Universal Definition of Myocardial Infarction. Among 1495 patients, the prevalence of NSTEMI was 17%. The optimal derived 0/1h-algorithm ruled-out NSTEMI with cMyC 0 h concentration below 10 ng/L (irrespective of chest pain onset) or 0 h cMyC concentrations below 18 ng/L and 0/1 h increase <4 ng/L. Rule-in occurred with 0 h cMyC concentrations of at least 140 ng/L or 0/1 h increase ≥15 ng/L. In the validation cohort (*n* = 663), the 0/1h-cMyC-algorithm classified 347 patients (52.3%) as ‘rule-out’, 122 (18.4%) as ‘rule-in’, and 194 (29.3%) as ‘observe’. Negative predictive value for NSTEMI was 99.6% [95% confidence interval (CI) 98.9–100%]; positive predictive value 71.1% (95% CI 63.1–79%). Direct comparison with the ESC hs-cTnT/I 0/1h-algorithms demonstrated comparable safety and even higher triage efficacy using the 0h-sample alone (48.1% vs. 21.2% for ESC hs-cTnT-0/1 h and 29.9% for ESC hs-cTnI-0/1 h; *P* < 0.001).

**Conclusion:**

The cMyC 0/1h-algorithm provided excellent safety and identified a greater proportion of patients suitable for direct rule-out or rule-in based on a single measurement than the ESC 0/1h-algorithm using hs-cTnT/I.

**Trial registration:**

ClinicalTrials.gov number, NCT00470587.

## Graphical Abstract

**Graphical Abstract:**
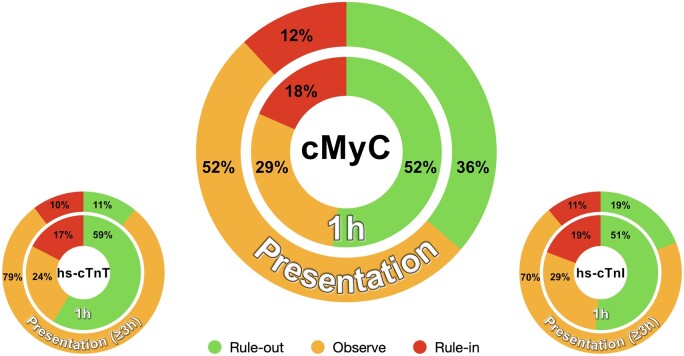

**Key points Question:** Could an algorithm employing cardiac myosin-binding protein C (cMyC) for triage of patients with chest pain accelerate rule-out and rule-in of myocardial infarction (MI)?

**Findings:** cMyC was able to triage a greater proportion of patients to early rule-out or rule-in of MI, while maintaining safety equivalent to hs-cTn-based algorithms (sensitivity ≥99%, negative predictive value ≥99.5% for rule-out).

**Meaning:** The use of cMyC instead or in addition to hs-cTnT/I could accelerate triage in the emergency department and potentially reduce length-of-stay by ruling-out more patients earlier.

## Introduction

Rapid diagnosis is a prerequisite for the timely initiation of potentially life-saving treatment in patients presenting with suspected acute myocardial infarction (AMI). Equally, *accurate* diagnosis and rule-out of AMI avoids potential side-effects from overtreatment of patients not requiring potent anti-platelet and anti-thrombotic medication. With the intent of extending the substantial improvements made in the initial management and consequent mortality of patients presenting with ST-elevation MI (STEMI) to those with non-ST-elevation MI (NSTEMI)^1^; the biochemical detection and quantification of acute cardiomyocyte necrosis is key. For this reason, the European Society of Cardiology (ESC) recommends the clinical use of rapid triage algorithms based on high-sensitivity cardiac troponin (hs-cTn) T/I concentrations.^2^ The ESC hs-cTnT/I 0/1h-algorithms rapidly rule-out and/or rule-in NSTEMI in patients presenting with acute chest discomfort to the emergency department (ED).^2^ This approach was made possible by the development and extensive clinical validation of hs-cTnT/I assays, which allow the detection and quantification of small amounts of cardiomyocyte necrosis.^3–8^

Recent real-world findings suggested that the clinical use of the ESC hs-cTnT 0/1h-algorithm is feasible, very safe, and associated with a substantially reduced time to ED discharge as compared to other algorithms including the ESC 0/3h-algorithm.^6,8–10^ However, even the most recently published ESC hs-cTnT/I 0/1h-algorithms provide opportunities for further improvements, e.g. by increasing the triage decisions that can be made on the immediate, 0 h, blood draw. Use of a more abundant, even more rapidly released biomarker, which is still a cardiac-restricted protein, such as cardiac myosin-binding protein C (cMyC); could potentially further improve the efficacy towards rule-out and/or rule-in.^11–16^

We, therefore, aimed to (i) derive and validate a 0/1h cMyC-algorithm to rapidly rule-out or rule-in NSTEMI, (ii) directly compare this to the well-established ESC hs-cTnT/I 0/1h-algorithms, and (iii) evaluate the incremental value of a dual marker approach integrating 0h-cMyC criteria into the established ESC hs-cTnT/I 0/1h-algorithms.

## Methods

In a prospective international diagnostic study enrolling patients presenting with suspected NSTEMI to the ED, venous blood samples were drawn at presentation (0 h) and after 1 h were measured for cMyC, hs-cTnT, and hs-cTnI.

### Study design and population

Advantageous Predictors of Acute Coronary Syndrome Evaluation (APACE) was a prospective international multicentre diagnostic study designed to advance the early diagnosis of NSTEMI.^17–20^ Adult patients presenting to the ED with acute chest discomfort possibly indicating NSTEMI were eligible for recruitment if the onset, or peak chest pain symptoms were within the preceding 12 h. Enrolment was independent of renal function, while patients with terminal kidney failure on chronic dialysis were excluded. For this analysis, the following patients were excluded: patients presenting with STEMI; patients with missing concentrations of cMyC at presentation or 1 h, and patients in whom the final diagnosis remained unclear after adjudication and at least one hs-cTnT level was elevated. A small proportion of patients did not have cMyC concentrations measured at presentation or 1h-repeat due to insufficient sample volume; baseline characteristics of patients with vs. without available cMyC measurements were comparable and are listed in Supplementary material online, *Table S1*. The protocol used for routine clinical assessment during patient enrolment has been described previously.^16^ The study was carried out according to the principles of the Declaration of Helsinki and approved by the local ethics committees. Written informed consent was obtained from all patients. T.K., R.T., T.N., J.B., P.A., M.R., K.W., and C.M. had full access to all the data in the study and take responsibility for its integrity and the data analysis. The authors designed the study, gathered, and analysed the data according to the STARD guidelines (Supplementary material online, *Table S1*) for studies of diagnostic accuracy, vouched for the data and analysis, wrote the paper, and decided to submit for publication.

### Adjudicated final diagnosis

Adjudication of the final diagnosis was performed centrally by two independent cardiologists according to the universal definition of MI using all available clinical information including cardiac imaging, serial hs-cTnT measurements, but blinded to cMyC.^21^ Two sets of data were used: first, all clinical data derived from routine clinical investigations including all available medical records—patient history, physical examination, results of laboratory testing including serial local (h)s-cTn, radiologic testing, ECG, echocardiography, cardiac exercise stress test, lesion severity, and morphology at coronary angiography—pertaining to the patient from the time of ED presentation to 90-day follow-up. Second, a study-specific assessment was collected, including 34 chest pain characteristics and serial hs-cTnT measurements to take advantage of the higher sensitivity and higher overall diagnostic accuracy offered by the more sensitive assays, as previously published.^7,17^ In situations of disagreement about the diagnosis, cases were reviewed and adjudicated in conjunction with a third cardiologist. In brief, NSTEMI was diagnosed when there was evidence of myocardial necrosis in association with a clinical setting consistent with myocardial ischaemia. Myocardial necrosis was diagnosed by at least one (h)s-cTn value above the 99th percentile together with a significant rise and/or fall. All other patients were classified into the categories of unstable angina (UA), cardiac non-coronary disease (e.g. tachyarrhythmias, perimyocarditis), non-cardiac chest pain, and symptoms of unknown origin. For follow-up and clinical endpoints, see Supplementary material online.

### Measurement of cMyC, hs-cTnT, and hs-cTnI

Blood samples for determination of cMyC, hs-cTnI, and hs-cTnT were collected into heparin plasma and serum tubes at presentation to the ED and serially thereafter (at time points 1 h, 2 h, 3 h, and 6 h). Serial sampling was discontinued when a diagnosis of NSTEMI was certain and treatment required patient transfer to the coronary care unit or catheter laboratory. After centrifugation, samples were frozen at −80°C until they were assayed in a blinded fashion in a dedicated core laboratory. cMyC was measured using the previously established, pre-commercial high-sensitivity assay on the Singulex Erenna platform that was performed by EMD Merck Millipore (Hayward, CA, USA).^22^ The assay has a lower limit of detection (LoD) of 0.4 ng/L and a lower limit of quantification (LoQ) of 1.2 ng/L with a ≤20% coefficient of variation at LoQ, ≤10% CV above 4.6 ng/L and, specifically, at 99th centile. For further details, please see Supplementary material online.

### Derivation and validation of the cMyC 0/1h-algorithm

The cMyC 0/1h-algorithm was developed in a derivation and validation design (random 1:1 split in cohort of patients with all biomarkers available; Supplementary material online, *Figure S1*). The concept of the cMyC 0/1h-algorithm was comparable to the ESC hs-cTnT/I 0/1h-algorithm (as per 2020 ESC guidelines^2^) as it allowed the rapid triage of patients with suspected NSTEMI towards rule-out, observe and rule-in based on cMyC concentrations obtained at presentation (0 h) and after 1 h (Supplementary material online, *Figure S2*). However, it differed in two important details: first, while the ESC hs-cTnT/I 0/1h-algorithm does not allow for direct rule-out based on a single hs-cTnT measurement in patients presenting very early (≤3 h) after chest pain onset due to concerns of delayed release of hs-cTnT/I in NSTEMI,^23^ the cMyC 0/1h-algorithm offered a direct rule-out option based on a single measurement irrespective of the time since chest pain onset (based on prior research describing an earlier rise and shorter time-to-peak concentration^11,13,16^) as well as favourable pilot data in patients presenting very early (≤3 h) after chest pain onset.^24^ Second, the ESC hs-cTnT/I 0/1h-algorithm considered dynamic changes as absolute, unsigned changes (not differentiating between rise or fall) between the 0h- and 1h-sample, whereas the cMyC 0/1h-algorithm considers absolute, signed changes (differentiating between rise or fall; based on prior insights into the release kinetics of cMyC).^11,13^

We derived and selected a cut-off combination fulfilling the pre-defined performance targets [negative predictive value (NPV) ≥99%, sensitivity ≥99% positive predictive value (PPV) ≥70%] in the derivation dataset. This particular cut-off combination was then applied to the validation dataset to test its performance. Prior publications have evaluated the cut-off concentrations in the presentation samples only.^16,24^ For integration of cMyC into ESC algorithms, see Supplementary material online.

### Statistical analysis

All data were expressed as medians [1st quartile; 3rd quartile] or means (standard deviation) for continuous variables (compared with the Mann–Whitney *U* test or Student’s *t*-test, respectively), and for categorical variables as numbers and percentages (compared with Pearson *χ*^2^). Hypothesis testing was two-tailed, and *P* values <0.05 were considered statistically significant. No adjustment for multiple comparisons was performed.

Rule-out safety of the 0/1h-algorithm was quantified by the NPV, sensitivity, and likelihood ratio (LR) for NSTEMI in the rule-out group. Accuracy of rule-in was quantified by the PPV, specificity, and LR for NSTEMI in the rule-in group. NPV and PPV were derived using the Bayes theorem and Jeffreys prior. Efficacy was quantified by the proportion of patients triaged to either rule-out or rule-in. Subgroup analyses were performed for patients presenting early (chest pain onset ≤3 h prior to ED presentation); as well as stratified by age groups, sex, and presence of renal disease.

Confidence intervals (CIs) of proportions, where appropriate, were computed using Wilson’s method. The McNemar test was used to compare sensitivity and specificity; NPV and PPV were compared using weighted generalized score statistics within the R package DTComPair.^25^

All statistical analyses were performed using R, version 3.6.1 (The R Foundation for Statistical Computing).

## Results

### Baseline demographics

Of 1495 patients with available cMyC measurements, 1326 had hs-cTnT, hs-cTnI, and cMyC results available at 0 h and 1 h (Supplementary material online, *Figure S1*). NSTEMI was the final diagnosis in 17%. Across the entire cohort, median age was 62 years [50; 75], 31% of patients were women and 37% had a prior history of CAD (*Table 1*, Supplementary material online, *Table S2*). Concentrations of cMyC, hs-cTnT, and hs-cTnI at 0 h and 1 h and absolute 0/1h-changes were significantly higher in NSTEMI compared to other causes of chest discomfort (Supplementary material online, *Table S3* and *Figures S3* and *S4*).

**Table 1:** Baseline demographics

Demographics	All patients (*N* = 1495)	No NSTEMI (*N* = 1236)	NSTEMI (*N* = 259)	*P* value^a^
Adjudicated NSTEMI	259 (17%)	0 (0%)	259 (100%)	NA
Female sex	464 (31%)	403 (33%)	61 (24%)	0.005
Age, years	62 [50; 75]	61 [49; 73]	72 [59; 80]	<0.001
Medical history				
Hypertension	943 (63%)	743 (60%)	200 (77%)	<0.001
Hyperlipidaemia	779 (52%)	610 (49%)	169 (65%)	<0.001
Diabetes mellitus	286 (19%)	211 (17%)	75 (29%)	<0.001
Current smoking	361 (24%)	292 (24%)	69 (27%)	0.341
History of smoking	560 (37%)	448 (36%)	112 (43%)	0.041
Previous revascularization (PCI or CABG)	430 (29%)	333 (27%)	97 (37%)	0.001
Coronary artery disease	547 (37%)	414 (33%)	133 (51%)	<0.001
Vital parameters				
Heart rate, b.p.m.	76 [66; 89]	75 [65; 89]	78 [69; 90]	0.027
Systolic blood pressure, mmHg	142 [126; 160]	142 [127; 159]	142 [126; 161]	0.592
Diastolic blood pressure, mmHg	82 [72; 92]	83 [72; 92]	80 [70; 91]	0.130
Laboratory results				
Estimated glomerular filtration rate, mL/min/1.73 m^2^^b^	85 [68; 101]	86 [70; 102]	73 [56; 95]	<0.001
cMyC 0 h, ng/L	16 [8; 49]	13 [7; 28]	211 [58; 689]	<0.001
cMyC 1h-change, ng/L^c^	0 [−2; 4]	0 [−2; 2]	33 [1; 145]	<0.001
hs-cTnT 0 h, ng/L	9 [5; 20]	7 [5; 13]	58 [26; 119]	<0.001
hs-cTnT 1h-change, ng/L^d^	1 [0; 2]	0 [0; 1]	8 [3; 22]	<0.001
hs-cTnI 0 h, ng/L	5 [2; 14]	4 [2; 8]	84 [18; 389]	<0.001
hs-cTnI 1h-change, ng/L^d^	1 [0; 2]	0 [0; 1]	27 [6; 124]	<0.001

Data are expressed as medians [1st quartile, 3rd quartile] or mean ± standard deviation, for categorical variables as *n* (%).

CABG, coronary artery bypass graft; IQR, interquartile range; NSTEMI, non-ST-segment elevation myocardial infarction as per gold-standard adjudication; PCI, percutaneous coronary intervention.

a *P* values for comparison NSTEMI group vs. no NSTEMI.

b Glomerular filtration rate was estimated using the modification of diet in renal disease (MDRD) formula.

c cMyC deltas are calculated as the signed value (differentiating between rise or fall).

d hs-cTn deltas are calculated as the unsigned value.

### Derivation of the cMyC 0/1h-algorithm

The optimal derived cMyC 0/1h-algorithm ruled-out NSTEMI with 0 h concentrations below 10 ng/L (irrespective of chest pain onset) or 0 h cMyC concentrations below 18 ng/L and a 0/1 h increase of <4 ng/L (Supplementary material online, *Figure S2*). Triage towards rule-in occurred with 0 h cMyC concentrations of at least 140 ng/L or a 0/1 h increase of at least 15 ng/L. Applying the 0/1 h cMyC-algorithm in the derivation cohort (*n* = 663), 325 patients (49.0%) could be classified as ‘rule-out’, 123 patients (18.6%) as ‘rule-in’, and 215 patients (32.4%) as ‘observe’ within 1 h. The NPV for NSTEMI in the rule-out group was 99.5% (95% CI 98.0–100), sensitivity was 99.1% (95% CI 97–100), and the PPV for NSTEMI in the rule-in group was 70.6% (95% CI 62.5–78.4; Supplementary material online, *Table S4*).

### Validation of the cMyC 0/1h-algorithm

Applying the derived cMyC 0/1h-algorithm in the validation cohort (*n* = 663, 91 NSTEMI), 36.2% (240) of patients could be ruled-out directly at presentation (based on 0h-sample only), and 52.3% (347) after the completed 0/1h-algorithm (*Figure 2*). Rule-out safety was very high and met the predefined target with an NPV of 99.6% (95% CI 98.9–100) and a sensitivity of 99.1% (95% CI 97–100; *Table 2* incl. performance in early presenters; misclassified patients see Supplementary material online, *Table S5*). There were no false-negatives amongst the subset of patients presenting within 3 h of chest pain onset (*n* = 250).

**Table 2:** Algorithm performance

	cMyC 0/1h-algorithm	ESC hs-cTnT 0/1h-algorithm	*P* ^a^	ESC hs-cTnI 0/1h-algorithm	*P* ^a^
Prevalence of NSTEMI	17% (same cohort)				
NPV	99.57% (98.88–100) EP: 100% (97.23–1)	99.87% (99.51–100)	0.317	99.56% (98.85–100)	0.714
Sensitivity	99.06% (97–100) EP: 100% (91.97–1)	100% (100–100)	0.317	99.12% (97.03–100)	1.000
PPV	71.14% (63.1–78.99) EP: 70.6% (57–81.29)	78.88% (71.4–86.08)	0.022	72.27% (64.47–79.86)	0.714
Specificity	93.6% (91.37–95.61) EP: 92.7% (88.33–95.54)	95.67% (93.85–97.26)	0.034	93.64% (91.52–95.67)	1.000
LR+	11.97 (8.77–17.46)	18.26 (12.62–28.75)		12.66 (9.29–18.32)	
LR−	0.01 (0–0.03)	0 (0–0.02)		0.01 (0–0.03)	
Proportion ruled-out					
Based on 0-h sample	240 (36.2%)	72 (10.9%)	<0.001	125 (18.9%)	<0.001
Based on 0/1-h samples	347 (52.3%)	390 (58.8%)	0.020	341 (51.4%)	0.784
Proportion ruled-in					
Based on 0-h sample	79 (11.9%)	68 (10.3%)	0.382	73 (11%)	0.666
Based on 0/1-h samples	122 (18.4%)	115 (17.4%)	0.667	127 (19.2%)	0.778
Overall efficacy					
Based on 0-h sample	319 (48.1%)	140 (21.1%)	<0.001	198 (29.9%)	<0.001
Based on 0/1-h samples	469 (70.7%)	505 (76.2%)	0.030	468 (70.6%)	0.904
Prevalence of NSTEMI in observational group	26 (13.4%)	23 (14.6%)	0.876	21 (10.8%)	0.522

Direct comparison of the performance of the cMyC 0/1h-algorithm to the established ESC hs-cTnT/I 0/1h-algorithms in the validation cohort (*n* = 663).

EP, early presenters; LR+, positive likelihood ratio; LR−, negative likelihood ratio; NPV, negative predictive value; NSTEMI, non-ST-elevation myocardial infarction; PPV, positive predictive value.

a *P* values for comparison cMyC to hs-cTn.

The cMyC 0/1h-algorithm classified 11.9% of patients as direct rule-in at 0 h, and 18.4% as rule-in after the completed algorithm. Accuracy was high and met the predefined target with a PPV of 71.1% (95% CI 63.1–79.0) and a specificity of 93.6% (95% CI 91.4–95.6). Overall, the cMyC 0/1h-algorithm assigned 70.7% of patients to either rule-out or rule-in categories, with 29.3% remaining in the observe zone with an NSTEMI prevalence of 13.4%.

### Direct comparison of the cMyC 0/1h-algorithm with the ESC hs-cTnT/I 0/1h-algorithms

**Figure 1:**
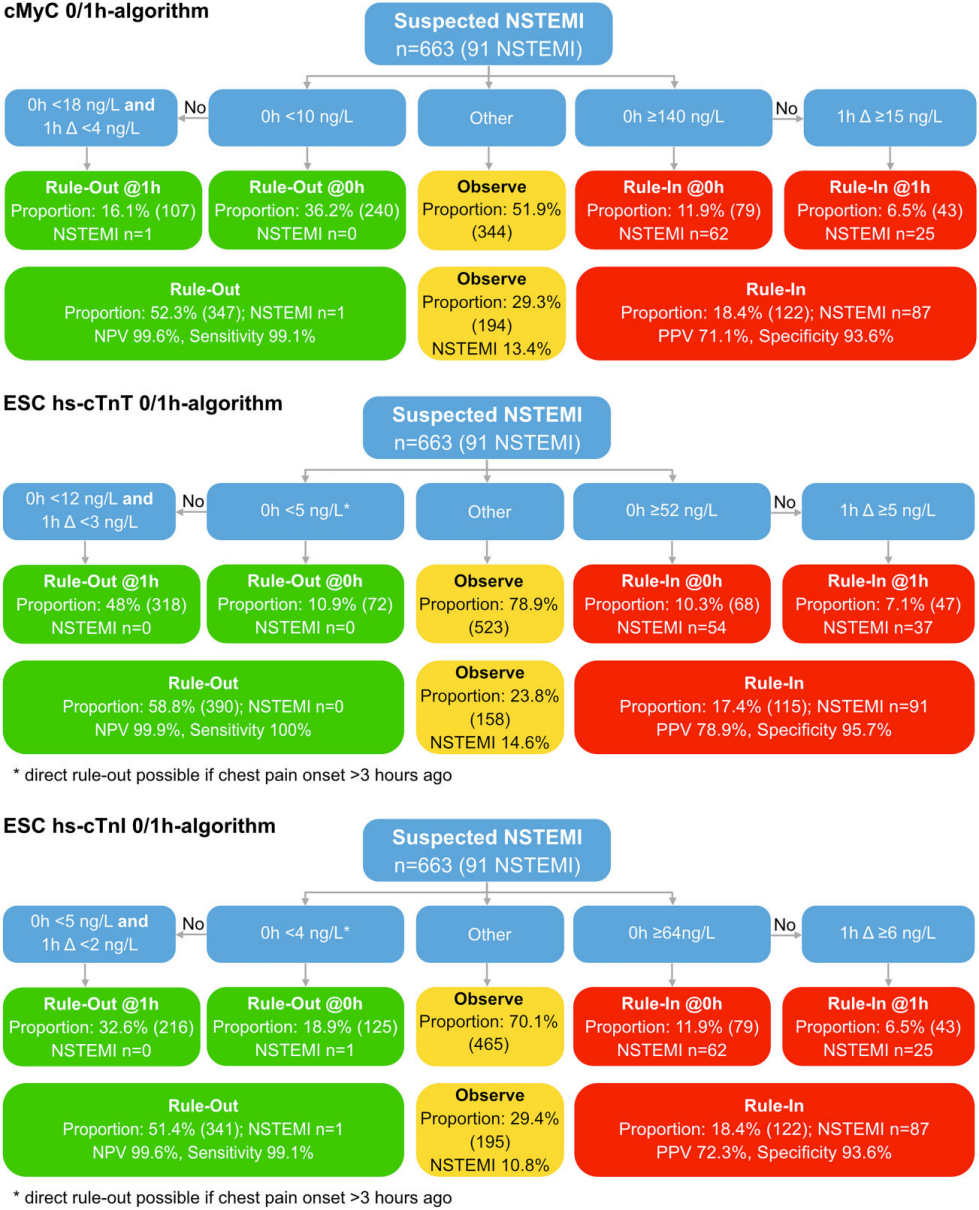
Comparison of cMyC and ESC hs-TnT/I 0/1h-algorithms: direct comparison of patient distribution in the validation cohort according to the cMyC 0/1h-algorithm and the ESC hs-cTnT/I 0/1h-algorithms. Final clinical adjudication of NSTEMI included hs-cTnT.

Overall, the performance of the cMyC 0/1h-algorithm was comparable to the ESC hs-cTnT/I 0/1h-algorithms (*Table 2* and *Figure 1*). Of note, the cMyC 0/1h-algorithm was more effective at direct rule-out based on the 0h-sample alone (cMyC 36.2% direct rule-out vs. 10.9% with hs-cTnT; vs. 18.9% with hs-cTnI; *P* < 0.001 both) and more effective at either direct rule-out or rule-in based on the 0h-sample alone (48.1% vs. 21.2% for ESC hs-cTnT-0/1 h and 29.9% for ESC hs-cTnI-0/1 h, both *P* < 0.001), but not after a completed 0/1h-algorithm [cMyC overall efficacy 70.7% vs. 76.2% with hs-cTnT (*P* = 0.03) and 70.6% with hs-cTnI [*P* = 0.904]). Predefined subgroup analysis confirmed the consistency of these findings (Supplementary material online, *Tables S6–S12* and *Figure S5*).

### Integrating 0h-cMyC criteria into the established ESC hs-cTnT/I 0/1h-algorithms

**Figure 2:**
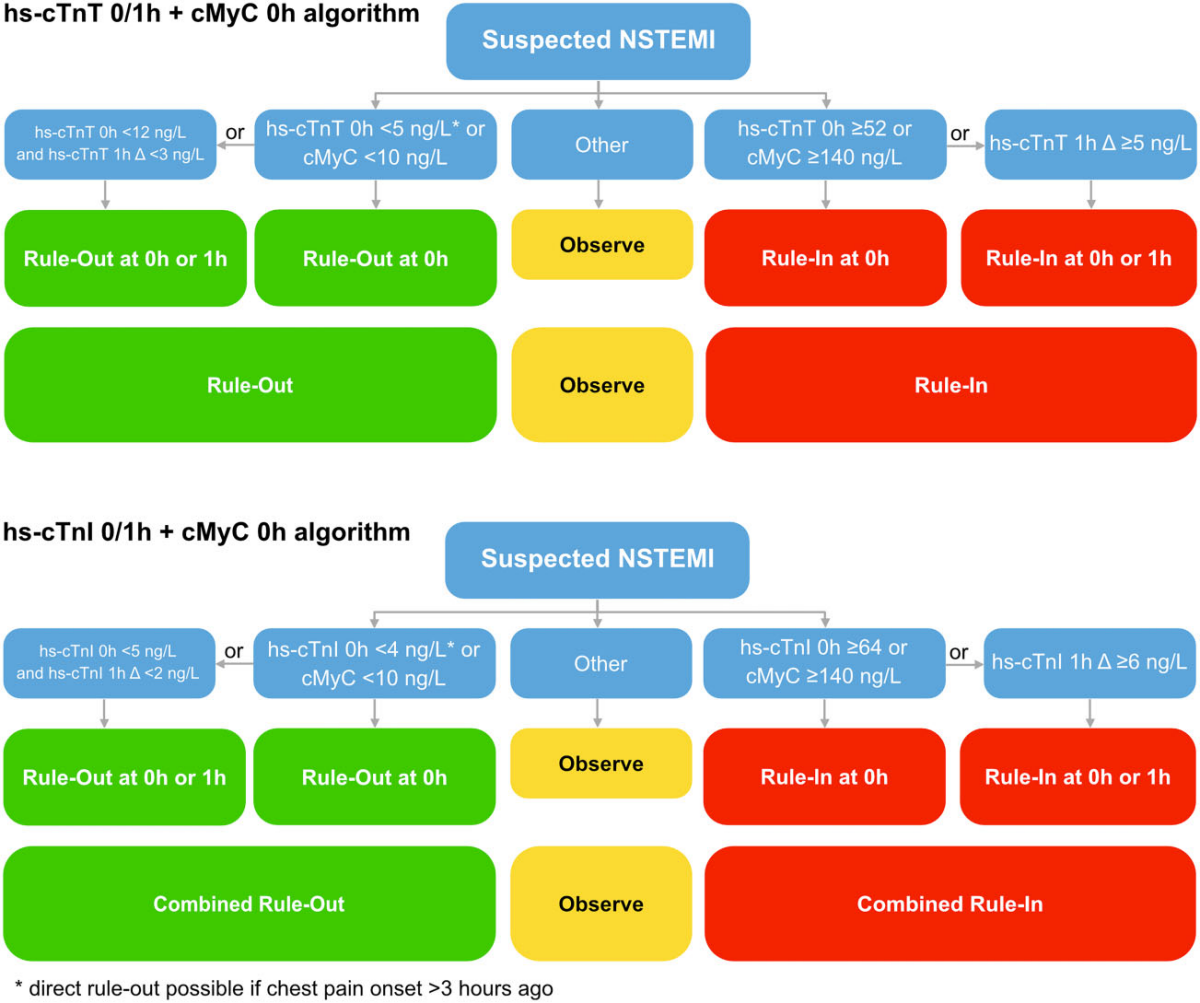
Dual-marker strategy: Concept outlining how the 0h cMyC concentration could be employed as a triage-booster when integrated into the established ESC 0/1h-algorithm.

Given the higher proportion of patients triaged towards rule-out with the 0h-cMyC criteria, the performance of a modified ESC hs-cTnT/I 0/1h-algorithms—using cMyC as an additional direct rule-out/rule-in criteria—was evaluated (*Figure 2*). This modified algorithm yielded comparable safety to the ESC hs-cTnT 0/1h-algorithm (NPV 99.9% both; sensitivity 100% both) but was inferior in terms of PPV (75.6% vs. 78.9%, *P* = 0.038) and specificity (94.6% vs. 95.6%, *P* = 0.014; Supplementary material online, *Figure S6*). There was no significant difference when comparing NPV, PPV, sensitivity, and specificity to the ESC hs-cTnI 0/1h-algorithm (Supplementary material online, *Figure S7*).

By adding the 0h-cMyC criteria to the triage process, the proportion of patients ruled-out and overall efficacy based on the 0-h samples was significantly increased: hs-cTnT + cMyC directly ruled-out 38.5% vs. 10.9% of patients for hs-cTnT alone (*P* < 0.001), overall efficacy at 0 h 51.7% vs. 21.1% (*P* < 0.001); hs-cTnI + cMyC direct rule-out 41.9% vs. 18.9% (*P* < 0.001), overall efficacy at 0 h 55.8% vs. 29.9% (*P* < 0.001; Supplementary material online, *Figures S6* and *S7* and *Tables S13* and *S14*).

### Prognostic utility for death at 30 days and 1 year

**Figure 3:**
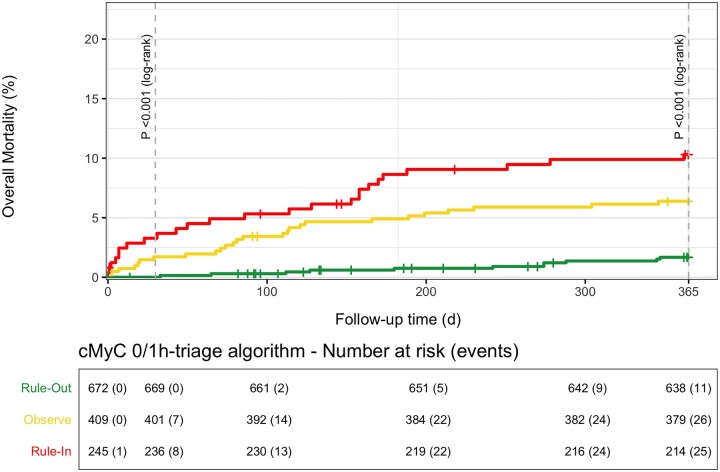
Mortality plot: cumulative event plot for the cMyC 0/1h-algorithm, with statistical comparison of event curves at 30 and 365 days with the log-rank tests; table displays number-at-risk and absolute number of events.

Beyond its diagnostic utility, the cMyC 0/1h-algorithm clearly delineates patients at varying degrees of risk of future events—*Figure 3*demonstrates a mortality plot stratified by triage category at 30-day and 1-year follow-up. Corresponding mortality data for hs-cTnT/I have been published before,^16^ but mortality plots have been reproduced to allow comparison (see Supplementary material online, *Figures S8* and *S9*).

## Discussion

This international multicentre study used central adjudication of the final diagnosis according to the fourth universal definition of MI and a derivation-validation design to contribute to a better understanding of the best possible clinical use of cMyC, a structural protein unique to cardiomyocytes that is more abundant and more rapidly released as compared to hs-cTnT/I.^11–16,26^ We report six major findings.

First, using the established concept of combining concentrations at ED presentation and absolute changes within 1 h, we were able to derive a cMyC 0/1h-algorithm achieving the predefined criteria of a NPV and sensitivity of at least 99% in the rule-out group and a PPV of at least 70% in the rule-in group in the validation cohort. Second, this algorithm enabled triage of three out of four patients with suspected NSTEMI based on the 0h and the 1h-sample. Third, the performance of the cMyC 0/1h-algorithm was comparable to that of the ESC hs-cTnT/I 0/1h-algorithms. Fourth, as an important differentiating feature, the cMyC 0/1h-algorithm triaged a substantially higher proportion of patients—an absolute increase of more than 25%—towards rule-out or rule-in based on the 0h-concentration alone. While this improvement diminishes after a completed 0/1h-algorithm, this ‘first shot’ benefit was at least in part due to a simplification of the single measurement rule-out pathway, which can be applied irrespective of the time interval from chest pain onset to ED presentation. In contrast, the single measurement rule-out pathway of the ESC hs-cTnT/I 0/1h-algorithms can only be applied in patients presenting 3 h or longer after chest pain onset. This differentiating feature might have allowed the algorithm to calibrate more closely to final outcomes, as the rapid release of cMyC following myocardial injury reduces the time to diagnosis by abolishing the need for repeat testing—rather than the biomarker exhibiting substantially different performance with respect to sensitivity and specificity. Fifth, similarly, integrating an alternative (‘or’) 0h-cMyC criteria into the established ESC hs-cTnT/I 0/1h-algorithms substantially increased (absolute increase of about 30%) the proportion of patients eligible for triage with the 0h-criteria. Sixth, beyond its diagnostic utility, the cMyC 0/1h-algorithm also accurately predicted all-cause mortality at 30 days and 1 year.

These findings extend and corroborate previous experimental and clinical studies on cMyC as a potential addition, or even alternative, to hs-cTnT/I in the early diagnosis of AMI.^11–16,27^ Pilot *invivo* studies have confirmed experimental studies that cMyC seems to be more rapidly released from injured cardiomyocytes in NSTEMI as compared to hs-cTnT/I.^13^ Among 1954 patients presenting with suspected AMI to the ED, discriminatory power for AMI, as quantified by the area under the receiver-operating characteristic curve (AUC), was comparable for cMyC (AUC, 0.924), hs-cTnT (AUC, 0.927), and hs-cTnI (AUC, 0.922) and superior to cTnI measured by a contemporary sensitivity assay (AUC, 0.909).^16^ The combination of cMyC with hs-cTnT or standard-sensitivity cTnI (but not hs-cTnI) led to an increase in AUC.

Increasing the proportion of patients eligible for safe rule-out of NSTEMI and possible early discharge from the ED using the 0h-cMyC criteria may have important medical and economic value. Arguably, the optimal approach could require the quantification of two cardiac biomarkers in the ED; a cardiac panel of hs-cTn and cMyC would hence provide the most benefit when implemented at the first blood draw to minimize the number of patients requiring ongoing observation and repeat blood testing. As chest pain and thereby suspected AMI is a very common complaint among patients presenting to EDs worldwide^27,28^—and responsible for ∼10–15 million attendances in the USA and Europe per year. Based on pragmatic assumptions, more than 1 million patients in the USA and Europe could benefit from earlier triage towards rule-out if the cMyC 0/1h-algorithm was used for initial assessment—this could translate into cost-savings exceeding €900 million/year (∼$1 billion). Dedicated cost-effectiveness analyses seem warranted.^29^

### Study limitations

This study has several limitations: first, cMyC measurements were performed on a research assay and still require migration onto a clinical laboratory platform for automated use as part of chest pain triage. Although transition to a clinically available, central laboratory-based analyser is in progress, and the assay will be calibrated against the same recombinant protein used in the research assay, it is clearly necessary to confirm identical results and thresholds in future, prospective studies. Despite its size, use of an internal validation cohort, and other methodological strengths (central adjudication by two independent cardiologists according to the universal definition of MI), external validation in another large multicentre study is required. Second, as a prospective diagnostic study, we cannot exactly quantify the clinical benefit associated with the use of cMyC as an alternative or addition to hs-cTnT/I. Third, we cannot comment on the performance of the cMyC 0/1h-algorithm among patients with terminal kidney failure on renal replacement therapy, because such patients were excluded from this study. Fourth, the performance of the cMyC 0/1h-algorithm in cohorts with lower prevalence of MI, as frequently found in chest pain populations outside Europe, requires further investigation. Fifth, the use of hs-cTnT as the adjudicating biomarker might have biased results towards that assay and would require comparison in a cohort adjudicated using, e.g. a hs-cTnI assay. This might also be responsible for remarkably similar efficacy when comparing algorithms not based on the adjudicating biomarker (cMyC and hs-cTnI).

## Conclusions

The cMyC 0/1h-algorithm provided excellent safety and identified a greater proportion of patients suitable for direct rule-out or rule-in based on a single measurement than the ESC 0/1h-algorithm using hs-cTnT/I.

## Supplementary material

Supplementary material is available at *European Heart Journal: Acute Cardiovascular Care* online.

## Supplementary Material

zuac007_Supplementary_Data

## Members of APACE Investigators

Michael Freese^1,2^, Paul David Ratmann^1,2^, Alexandra Prepoudis^1,2^, Danielle M. Gualandro^1,2^, Nicolas Geigy^2,3^, Tobias Reichlin^2,4^, Katharina Rentsch^5^, Mario Maier^1,2^, Valentina Troester^1,2^, Juliane Gehrke^1,2^, Tania Coscia^1,2^, Noemi Glarner^1,2^, Hadrien Schoepfer^1,2^, Michael Buechi^1,2^, Joan Walter^1,2^, Ana Yufera Sanchez^1,2^, Christian Puelacher^1,2^, Jeanne du Fay de Lavallaz^1,2^, Alessandra Sanzione^1,2^, Ibrahim Schäfer^1,2^, Petra Hillinger^1,2^, Beatriz López^6^, Esther Rodriguez Adrada^7^, Piotr Muzyk^2,8^, Beata Morawiec^2,8^, Jiri Parenica^2,9^, Eva Ganovská^2,9^, Jens Lohrmann^1,10^, Andreas Buser^11^, Arnold von Eckardstein^12^, Roland Bingisser^10^, and Christian Nickel^10^

^1^Department of Cardiology and Cardiovascular Research Institute Basel (CRIB), University Hospital Basel, University of Basel, Switzerland; ^2^GREAT Network; Emergency Department, Kantonsspital Liestal, Liestal, Switzerland; ^4^Department of Cardiology, Inselspital, Bern University Hospital, University of Bern, Bern, Switzerland; ^5^Department of Laboratory Medicine, University Hospital Basel, Basel, Switzerland; ^6^Servicio de Urgencias, Hospital Clínic, Barcelona, Spain; ^7^Emergency department, Hospital Clinico San Carlos, Madrid, Spain; ^8^2nd Department of Cardiology, Medical University of Silesia, Zabrze, Poland; ^9^Department of Cardiology, University Hospital Brno, Brno, Czech Republic and Medical Faculty, Masaryk University, Brno, Czech Republic; ^10^Emergency Department, University Hospital Basel, University of Basel, Basel, Switzerland; ^11^Blood Transfusion Centre, Swiss Red Cross, Basel, Switzerland and Department of Hematology, University Hospital Basel, University of Basel, Basel, Switzerland; ^12^Department of Laboratory Medicine, University Hospital Zurich, Zurich, Switzerland.
