# Supplementary material for: A 0/1h-algorithm using cardiac myosin-binding protein C for early diagnosis of myocardial infarction
Source: Eur Heart J Acute Cardiovasc Care. 2022 Feb 12;11(4):325–35. doi: 10.1093/ehjacc/zuac007 (PMC9173679; doi:10.1093/ehjacc/zuac007)
Supplement: zuac007_Supplementary_Data [file zuac007_supplementary_data.docx]

**A 0/1h-algorithm using cardiac myosin-binding protein C for early diagnosis of myocardial infarction - online supplement**

Trial Registration: **ClinicalTrials.gov number, NCT00470587**

**Abbreviations**

ED – Emergency department

AMI – Acute Myocardial Infarction

NSTEMI – Non-ST elevation Myocardial Infarction

ECG – Electrocardiography

cTn – Cardiac troponin

hs-cTn – High-sensitivity cardiac troponin

cMyC – cardiac Myosin-binding Protein C

eGFR – Estimated glomerular filtration rate

NPV – Negative predictive value

PPV – Positive predictive value

IQR – Interquartile range

# Supplement

## Supplemental methods

### Routine clinical assessment

All patients underwent a clinical assessment that included medical history, physical examination, 12-lead ECG, pulse oximetry, standard blood test, and chest radiography according to local protocols and in accordance with the guidelines of the European Society of Cardiology (ESC). Levels of cTn were measured at presentation and serially thereafter as long as clinically indicated. Treatment of patients was left to discretion of the attending physician.

### Adjudication of the final diagnosis

NSTEMI was defined and cTn levels interpreted as recommended in current guidelines. In brief, NSTEMI was diagnosed when there was evidence of myocardial necrosis with a significant rise and/or fall in a clinical setting consistent with myocardial ischemia. Patients with NSTEMI were further subdivided into type 1 MI (primary coronary events) and type 2 MI (ischemia due to increased demand or decreased supply, for example tachyarrhythmia or hypertensive crisis).

The adjudication of final diagnoses was performed centrally in the core lab (University Hospital Basel) for all patients incorporating levels of hs-cTnT (see test characteristics above). More specifically, two independent cardiologists not directly involved in patient care reviewed all available medical records (including patient history, physical examination, results of laboratory testing including (hs-)cTnT levels, radiologic testing, ECG, echocardiography, cardiac exercise test, lesion severity and morphology in coronary angiography, discharge summary) pertaining to the patient from the time of ED presentation to 90-day follow-up. Late samples were available for adjudication of final diagnosis in all patients. In general, serial sampling was performed until at least 6h after presentation to the ED or onset of chest. In situations of diagnostic disagreement, cases were reviewed and adjudicated in conjunction with a third cardiologist. While discharge diagnoses often were correct and in agreement with the final adjudicated diagnosis, there were also cases where those diagnoses needed to be revised, most often because more information became available from medical testing during early follow-up, and more rarely, because the discharge diagnosis was not in agreement with the Universal Definition of MI.

The 99^th^ percentile (14 ng/L) was used as cut-off for myocardial necrosis. Absolute cTn changes were used to determine significant changes based on the diagnostic superiority of absolute over relative changes. Based on studies of the biological variation of cTn(1, 2) as well as on data from previous chest pain cohort studies(3, 4), a significant absolute change was defined as a rise or fall of at least 10 ng/L within six hours, or, in an assumption of linearity, as an absolute change of 6 ng/L within three hours. Predefined alternative diagnoses included ‘unstable angina’ (UA), ‘Cardiac symptoms of origin other than coronary artery disease’ and ‘non-cardiac chest pain’.

### Clinical Care: The (hs-)cTn assays and cut-off levels used for local clinical care

Routine clinical care comprised five different cTn assays at the different hospitals and at the different recruitment periods. The cTn assays used clinically in most of the participating institutions changed during the study from a contemporary cTn assay to the hs-cTnT assay. In order to take advantage of the higher sensitivity and higher overall diagnostic accuracy offered by the hs-cTnT assay, patients were adjudicated using the hs-cTnT values in all patients. In patients in whom clinically a contemporary cTn assay was used, the concentrations of both, contemporary cTn and hs-cTnT, were available for the adjudication. Where hs-cTnT was the only clinically available biomarker, only hs-cTnT results were available for adjudication.

The following conventional cTn assays were used: For the Roche cTnT 4^th^ generation assay, the 10% CV level is 0.035 µg/L. The laboratories of the participating sites reported only two decimals; therefore 0.04 µg/L was used as a cut-off for myocardial necrosis. In order to fulfil the criteria of a significant change (30% of 99^th^ percentile or 10% CV level), a patient would e.g. need to have a level of <0.01 µg/L at presentation and 0.04 µg/L at 6h. A patient would also qualify if the first level is 0.02 µg/L and the second 0.04 µg/L. A patient would not fulfil the criteria if the first level is 0.03 µg/L and the second is 0.04 µg/L. If the first level is 0.04 µg/L, the second level needs to be at least 0.06 µg/L.

For Elecsys hs-cTnT measured clinically, the same change criteria were applied as for hs-cTnT measured from the study blood samples.

### Central adjudication: Definition of rise and/or fall in high-sensitivity cardiac troponin T (hs-cTnT)

Absolute changes in hs-cTnT were used to determine significant changes based on the diagnostic superiority of absolute over relative changes. Based on studies of the biological variation of cTn as well as on data from previous chest pain cohort studies, a significant absolute change was defined as a rise or fall of at least 10 ng/L within 6 hours or an absolute change of 6 ng/L within 3 hours. If later clinical samples (e.g., at 24, 48, or 72 hours) revealed a lower hs-cTnT level than that measured during the period of sampling in the ED, the later level was considered the true baseline level for the calculation of the change criteria.

## Follow-up and clinical endpoints

Patients were contacted 3, 12 and 24 months after discharge by telephone calls or in written form. Additionally, information regarding death during follow up was obtained from the patient’s hospital notes, the family physician’s records and the national registry on mortality. The primary diagnostic endpoint was NSTEMI (type 1 and 2) at presentation to the ED. The co-primary prognostic endpoints were all-cause mortality at 30 days and one year.

### Measurement of cardiac myosin-binding protein C

We have previously described the creation, biophysical selection and organ specificity of mouse monoclonal antibodies recognising cardiac-restricted epitopes within the N-terminus of cMyC. Two of these antibodies, 1A4 and 3H8, were used to create a sensitive sandwich immunoassay. In brief, magnetic microparticles (MPs) for capture were prepared by binding 25 μg of mouse monoclonal (1A4) per mg of MPs. The coated MPs were diluted in assay buffer (proprietary mix with custom 450 mM NaCl and 0.5% Triton X-100) to 100 µg/mL. Due to sample volume constraints, serum, plasma or analyte (recombinant C0C2 domain of cMyC) were diluted 2.2-fold with standard diluent and 100 µL added per well of a 96-well assay plate. Samples or standards were then exposed to 100 µL of coated MPs and agitated for 2 hours at 25°C. MPs were retained via a magnetic bed with unbound material removed in a single wash step. Fluorescently-labelled mouse monoclonal (3H8) detection antibody was diluted in assay buffer to 100 ng/mL. To each well, 20 µL of detection antibody was added and the MPs agitated for 1 hour at 25°C, retained via a magnetic bed and then washed 4 times to remove any unbound detection reagent. The MPs were then transferred to a new plate and all buffer was aspirated. The MPs were then exposed to 20 µL/well of elution buffer B for 5 minutes at 25°C before transfer to a 384-well plate containing 10 µL/well of neutralization buffer D. Fluorescent label was then detected by single molecule counting using the Erenna system with a dwell time of 60s per well. Three signal outputs were obtained from the Erenna System: Detected Events (DEs; low end signal), Event Photons (EPs; low end and higher end signal), and Total Photons (TPs; high end signal).

The assay has a lower Limit of Detection (LoD) of 0.4 ng/L and a lower Limit of Quantification (LoQ) of 1.2 ng/L with a ≤20% coefficient of variation at LoQ, ≤10% CV above 4.6 ng/L and, specifically, at 99^th^ centile. The 99^th^ percentile cut-off point determined previously (in patients without obstructive coronary artery disease (CAD) on invasive angiography) is 87 ng/L. Assay precision is not affected by freeze/thaw cycles, and results are closely correlated across different matrices (serum, lithium heparin, K2 EDTA) (5).

The hs-cTnT assay was measured on the Elecsys 2010 analyzer (Roche Diagnostics, Rotkreuz, Switzerland). The limit of blank and LoD were determined to be 3 and 5 ng/L, respectively. The 99th-percentile of a healthy reference population was reported at 14 ng/L with an imprecision corresponding to 10% CV at 13 ng/L (6). This study does not include any measurements with hs-cTnT lots that required the revision of the calibration curve.(7) The hs-cTnI assay (ARCHITECT STAT high-sensitivity troponin I, Abbott Laboratories, IL, USA) was measured on an Architect analyzer and has a 99th percentile concentration of 26.2 ng/L with a corresponding coefficient of variation (CV) of <5% and a limit of detection (LoD) of 1.9 ng/L (8).

## Integration of 0h-cMyC criteria into the ESC hs-cTnT/I 0/1h-algorithms

To test the possible incremental value of a dual marker approach integrating both hs-cTnT/I and cMyC, the established ESC hs-cTnT/I-0/1h-algorithms were extended by the integration of a single cMyC cut-off concentrations at 0h (9).

## Healthcare economic assessment – foundation for quoted cost savings achievable

According to NHS data, each day in hospital costs £200-400 (10, 11). By assuming an average cost of £300 per day in hospital, a calculation including an analysis of efficiency savings by using more rapid rule-out testing and thus reducing the overall length of stay can be performed. The following assumptions apply to the mathematical modelling of the Length of Stay (LOS): patients in immediate rule-out group (absolute numbers) can be discharged within 4 hours, patients in observe group are admitted for at least 24 hours (1 overnight-stay), of which 50% go on to be admitted for 3 days. Patients in the rule-in group are admitted for a mean of 3 days.

Costs for ED stay in the immediate rule-out group was extrapolated from work by Goodacre et al (12): Nursing 3h: £45, Medical time 45mins: £15, CXR: £6, Hospital overheads 4hrs: £15; Total: £81 per episode; Costs for ED + overnight stay: (ED costs - hospital overheads) + £300 for one day in hospital = £366 (the initial 3 hours are assumed to be more labour- and investigation-intense), every subsequent day £300/24hrs.

In tables M1 and M2 (reproduced from (13)), we provide an overview of the cost (savings) associated with e.g. hs-cTnI vs cMyC testing, on the basis of previous work evaluating cost-effectiveness of biomarker testing in the hospital environment. These tables were modelled on the use of cardiac biomarkers in the Emergency Department of a tertiary cardiac centre in central London and showcase a potential cost difference depending on whether a new biomarker (cMyC) was to be priced at a similar (M2) or higher cost (M1) per assay. Cost justification for the biomarker test can be provided upon request.

While the triage distribution is different to the results in the *paper*, the absolute difference between the biomarkers is ~17% which reflects the relative performance of hs-cTnT/I when compared to cMyC (see table 2, main paper). Extrapolating the findings to a population of 10-15 million individuals presenting with suspected AMI to Emergency Departments in the US and Europe, the potential savings range between £966 million and £1 billion (not accounting for country-specific differences in cost).

|  | **Conventional test – cTnI** | **Cost: £7.18** | **novel test – cMyC** | **Cost: £16.59** | **Costs of new test** |
| --- | --- | --- | --- | --- | --- |
| **Tests annually** | **7800** | **£56,004.00** |  | **£129,438.84** | **£73,434.84** |
| **Immediate Rule-out** | **15%** | **1170** | **32%** | 2496 |  |
| Cost estimate | **£81.00** | **£94,770.00** |  | **£202,176** | **£107,406.00** |
| **Observe group** | **70%** | **5460** | **52%** | 4056 |  |
| Cost estimate #1 | **£366.00** | **£999,180.00** |  | £742,248.00 |  |
| Cost estimate #2 | **£966.00** | **£2,637,180.00** |  | £1,959,048.00 | **£(935,064.00)** |
| **Rule-in** | **15%** | **1170** | **15%** | 1170 |  |
| Cost estimate | **£966.00** | **£1,130,220.00** |  | **£1,130,220.00** | **£-** |
| **Total costs** |  |  |  |  | **£(754,223.16)** |

Table M1: Cost estimation for Troponin I, assuming 17% higher immediate discharge; reproduced from Kaier T (13)

|  | **Conventional test – cTnI** | **Cost: £7.18** | **novel test – cMyC** | **Cost: £7.18** | **Costs of new test** |
| --- | --- | --- | --- | --- | --- |
| **Tests annually** | **7800** | **£56,004.00** |  | **£56,004.00** | **£-** |
| **Immediate Rule-out** | **15%** | **1170** | **32%** | 2496 |  |
| Cost estimate | **£81.00** | **£94,770.00** |  | **£202,176** | **£107,406.00** |
| **Observe group** | **70%** | **5460** | **52%** | 4056 |  |
| Cost estimate #1 | **£366.00** | **£999,180.00** |  | £742,248.00 |  |
| Cost estimate #2 | **£966.00** | **£2,637,180.00** |  | £1,959,048.00 | **£(935,064.00)** |
| **Rule-in** | **15%** | **1170** | **15%** | 1170 |  |
| Cost estimate | **£966.00** | **£1,130,220.00** |  | **£1,130,220.00** | **£-** |
| **Total costs** |  |  |  |  | **£(827,658.00)** |

Table M2: Cost estimation for Troponin I, assuming 17% higher immediate discharge, cMyC at same price as Troponin; reproduced from Kaier T (13)

## Supplemental tables

|  | **Section & Topic** | **No** | **Item** | **Reported on page #** |
| --- | --- | --- | --- | --- |
|  |  |  |  |  |
|  | **TITLE OR ABSTRACT** |  |  |  |
|  |  | **1** | Identification as a study of diagnostic accuracy using at least one measure of accuracy  (such as sensitivity, specificity, predictive values, or AUC) | 3 |
|  | **ABSTRACT** |  |  |  |
|  |  | **2** | Structured summary of study design, methods, results, and conclusions  (for specific guidance, see STARD for Abstracts) | 3 |
|  | **INTRODUCTION** |  |  |  |
|  |  | **3** | Scientific and clinical background, including the intended use and clinical role of the index test | 5-6 |
|  |  | **4** | Study objectives and hypotheses | 5-6 |
|  | **METHODS** |  |  |  |
|  | *Study design* | **5** | Whether data collection was planned before the index test and reference standard  were performed (prospective study) or after (retrospective study) | 6 |
|  | *Participants* | **6** | Eligibility criteria | 6 |
|  |  | **7** | On what basis potentially eligible participants were identified  (such as symptoms, results from previous tests, inclusion in registry) | 6 |
|  |  | **8** | Where and when potentially eligible participants were identified (setting, location and dates) | 6-7 |
|  |  | **9** | Whether participants formed a consecutive, random or convenience series | 7-8 |
|  | *Test methods* | **10a** | Index test, in sufficient detail to allow replication | 7-8 |
|  |  | **10b** | Reference standard, in sufficient detail to allow replication | 8 |
|  |  | **11** | Rationale for choosing the reference standard (if alternatives exist) | 8 |
|  |  | **12a** | Definition of and rationale for test positivity cut-offs or result categories  of the index test, distinguishing pre-specified from exploratory | 8-9 |
|  |  | **12b** | Definition of and rationale for test positivity cut-offs or result categories  of the reference standard, distinguishing pre-specified from exploratory | 8-9 |
|  |  | **13a** | Whether clinical information and reference standard results were available  to the performers/readers of the index test | 7-9 |
|  |  | **13b** | Whether clinical information and index test results were available  to the assessors of the reference standard | 7-9 |
|  | *Analysis* | **14** | Methods for estimating or comparing measures of diagnostic accuracy | 10 |
|  |  | **15** | How indeterminate index test or reference standard results were handled | 8-9 |
|  |  | **16** | How missing data on the index test and reference standard were handled | 6-8 |
|  |  | **17** | Any analyses of variability in diagnostic accuracy, distinguishing pre-specified from exploratory | 8 |
|  |  | **18** | Intended sample size and how it was determined | 8 |
|  | **RESULTS** |  |  |  |
|  | *Participants* | **19** | Flow of participants, using a diagram | Suppl figure S1, p8 |
|  |  | **20** | Baseline demographic and clinical characteristics of participants | 25 |
|  |  | **21a** | Distribution of severity of disease in those with the target condition | 25 |
|  |  | **21b** | Distribution of alternative diagnoses in those without the target condition | 25 |
|  |  | **22** | Time interval and any clinical interventions between index test and reference standard | 25 |
|  | *Test results* | **23** | Cross tabulation of the index test results (or their distribution)  by the results of the reference standard | 25 |
|  |  | **24** | Estimates of diagnostic accuracy and their precision (such as 95% confidence intervals) | 26 |
|  |  | **25** | Any adverse events from performing the index test or the reference standard | NA |
|  | **DISCUSSION** |  |  |  |
|  |  | **26** | Study limitations, including sources of potential bias, statistical uncertainty, and generalisability | 14-15 |
|  |  | **27** | Implications for practice, including the intended use and clinical role of the index test | 14-16 |
|  | **OTHER INFORMATION** |  |  |  |
|  |  | **28** | Registration number and name of registry | 4 |
|  |  | **29** | Where the full study protocol can be accessed | 4 |
|  |  | **30** | Sources of funding and other support; role of funders | 18-19 |
|  |  |  |  |  |

Table S1 – STARD checklist

| Demographics – missing vs present cMyC data at baseline (0h) and 1h repeat | | | |
| --- | --- | --- | --- |
| cMyC at 0h+1h | **Missing N=1503** | **Present N=1326** | **P value*** |
| Adjudicated NSTEMI | 268 (18%) | 226 (17%) | 0.617 |
| Male | 1021 (68%) | 907 (68%) | 0.82 |
| Age, years | 60 [49;74] | 63 [50;75] | 0.005 |
| Past Medical History |  |  |  |
| Hypertension | 904 (60%) | 834 (63%) | 0.144 |
| Hyperlipidaemia | 728 (48%) | 685 (52%) | 0.094 |
| Diabetes mellitus | 260 (17%) | 255 (19%) | 0.201 |
| Current smoking | 407 (27%) | 313 (24%) | 0.038 |
| History of smoking | 523 (35%) | 504 (38%) | 0.083 |
| Previous revascularisation (CABG or PCI) | 411 (27%) | 379 (29%) | 0.49 |
| Coronary artery disease | 499 (33%) | 483 (36%) | 0.079 |
| Observations |  |  |  |
| Heart rate, beats/min | 77 [66;90] | 75 [66;89] | 0.107 |
| Systolic blood pressure, mm Hg | 140 [126;157] | 142 [127;160] | 0.074 |
| Diastolic blood pressure, mm Hg | 82 [73;92] | 82 [71;91] | 0.293 |
| Laboratory Results |  |  |  |
| Estimated glomerular filtration rate, ml/min/1.73m2† | 85 [70;101] | 85 [68;101] | 0.58 |
| hs-cTnT 0h | 8 [5;20] | 9 [5;20] | 0.26 |
| hs-cTnT 1h-change | 0 [0;2] | 1 [0;2] | 0.09 |
| hs-cTnI 0h | 5 [2;19] | 5 [2;14] | 0.3 |
| hs-cTnI 1h-change | 1 [0;5] | 1 [0;2] | 0.828 |

Table S2 – Demographics for comparison of patients with missing vs present cMyC 0+1h concentrations; * p values for comparison NSTEMI group versus all other diagnoses; data are expressed as medians [1^st^ quartile, 3^rd^ quartile] or means ± standard deviation, for categorical variables as numbers (percentages); NSTEMI = Non-ST elevation Myocardial Infarction as per gold-standard adjudication; IQR = Interquartile Range; CABG = Coronary Artery Bypass Graft; PCI = Percutaneous Coronary Intervention; † glomerular filtration rate was estimated using the Modification of Diet in Renal Disease (MDRD) formula

## Distribution of biomarker concentrations for 0h, 1h samples and delta-change

| Diagnosis | cMyC 0h | hs-cTnT 0h | p* | hs-cTnI 0h | p* | n |
| --- | --- | --- | --- | --- | --- | --- |
| NSTEMI | 213 [59; 652] | 56 [23; 116] | <0.001 | 87 [20; 419] | <0.001 | 226 |
| CNC | 31 [12; 79] | 14 [7; 31] | <0.001 | 9 [4; 32] | <0.001 | 189 |
| UA | 21 [13; 44] | 11 [7; 17] | <0.001 | 6 [3; 13] | <0.001 | 138 |
| NCCP | 10 [6; 19] | 6 [5; 10] | <0.001 | 3 [2; 5] | <0.001 | 722 |
| unknown | 11 [7; 16] | 6 [5; 9] | <0.001 | 3 [2; 4] | <0.001 | 51 |
| Diagnosis | cMyC 1h | hs-cTnT 1h | p* | hs-cTnI 1h | p* | n |
| NSTEMI | 304 [77; 899] | 67 [30; 148] | <0.001 | 138 [29; 547] | <0.001 | 226 |
| CNC | 30 [12; 99] | 15 [8; 31] | <0.001 | 11 [4; 42] | <0.001 | 189 |
| UA | 22 [14; 46] | 12 [7; 17] | <0.001 | 7 [4; 14] | <0.001 | 138 |
| NCCP | 10 [6; 18] | 6 [5; 10] | <0.001 | 3 [2; 5] | <0.001 | 722 |
| unknown | 13 [7; 17] | 5 [5; 9] | <0.001 | 3 [2; 5] | <0.001 | 51 |
| Diagnosis | cMyC delta | hs-cTnT delta | p* | hs-cTnI delta | p* | n |
| NSTEMI | 37 [2; 156] | 8 [1; 22] | <0.001 | 20 [2; 117] | 0.089 | 226 |
| CNC | 0 [-2; 8] | 0 [0; 2] | 0.064 | 0 [0; 4] | 0.547 | 189 |
| UA | 1 [-2; 3] | 0 [-1; 1] | 0.103 | 0 [0; 1] | 0.108 | 138 |
| NCCP | 0 [-1; 1] | 0 [0; 0] | 0.916 | 0 [0; 0] | 0.144 | 722 |
| unknown | 0 [-2; 3] | 0 [-1; 0] | 0.431 | 0 [0; 0] | 0.462 | 51 |

Table S3 – Biomarker distribution per time-point and for 1h-delta change value; all values in ng/L; NSTEMI = Non-ST elevation Myocardial Infarction; CNC = Cardiac non-coronary; UA = Unstable Angina; NCCP = Non-cardiac Chest Pain; * p = direct comparison between medians within groups

## cMyC 0/1h-algorithm performance in derivation cohort

| n=663 (87 NSTEMI) | cMyC | hs-cTnT | p* |
| --- | --- | --- | --- |
| NPV | 99.54% (98.8-100) | 99.87% (99.48-100) | 0.317 |
| Sensitivity | 99.11% (97.03-100) | 100% (100-100) | 0.317 |
| PPV | 70.56% (62.51-78.43) | 76.09% (68.24-83.68) | 0.071 |
| Specificity | 93.48% (91.41-95.29) | 95.13% (93.27-96.92) | 0.061 |
| LR+ | 11.89 (8.7-17.11) | 15.85 (11.1-24.23) |  |
| LR- | 0.01 (0-0.03) | 0 (0-0.02) |  |
| Proportion ruled-out |  |  |  |
| Based on 0-hour sample | 31.4% | 8.1% | <0.001 |
| Based on 0/1-hour samples | 49.0% | 56% | 0.013 |
| Proportion ruled-in |  |  |  |
| Based on 0-hour sample | 13.4% | 12.2% | 0.565 |
| Based on 0/1-hour samples | 18.6% | 17.2% | 0.566 |
| Overall efficacy |  |  |  |
| Based on 0-hour sample | 44.8% | 20.36% | <0.001 |
| Based on 0/1-hour samples | 67.6% | 73.2% | 0.030 |
| Prevalence of NSTEMI in observe group | 11.2% | 14.0% | 0.479 |

Table S4 – Direct comparison of the performance of the cMyC 0/1h-algorithm to the established ESC hs-cTnT 0/1h-algorithm in derivation cohort; NPV = Negative Predictive Value; PPV = Positive Predictive Value; LR- = Negative Likelihood Ratio; LR+ = Positive Likelihood Ratio; NSTEMI = Non-ST elevation Myocardial Infarction; * p values for comparison cMyC to hs-cTnT

| Validation cohort – ‘missed’ myocardial infarcts | | | | |  |  |  |  |  | Triage | | | cMyC | | hs-cTnT | | hs-cTnI | | | |
| --- | --- | --- | --- | --- | --- | --- | --- | --- | --- | --- | --- | --- | --- | --- | --- | --- | --- | --- | --- | --- |
| Age | Gender | Chol | DM | Smoking history | Previous MI | CAD | GFR | Chest pain to blood draw (h) | Adjudication | hs-cTnT | hs-cTnI | cMyC | 0h | 1h | 0h | 1h | 0h | | 1h |  |
| 63 | female | yes | diet | no | yes | yes | 75 | 2 | Type 1 MI | Rule-In | Rule-In | Rule-Out | 12 | 15 | 16 | 5 | 12 | | 25 |  |
| 93 | female | yes | no | no | no | yes | 34 | 9 | Type 1 MI | Observe | Rule-Out | Rule-In | 47 | 67 | 41 | 38 | | 4 | 2 | |

Table S5 – Overview of patients missed by any biomarker during triage process; Chol = Hypercholesterolaemia; DM = Diabetes mellitus; MI = Myocardial Infarction; GFR = glomerular filtration rate was estimated using the Modification of Diet in Renal Disease (MDRD) formula

## Subgroup analysis

The full dataset containing all timepoints (cMyC 0+1h, hs-cTnT 0+1h, hs-cTnI 0+1h; total n=1326) was grouped according to the analysis plan – see tables below.

| All patients | | | | | |
| --- | --- | --- | --- | --- | --- |
| n=1326 (178) | **cMyC** | **hs-cTnT** | **p*** | **hs-cTnI** | **p†** |
| NPV | 99.63% (99.17-99.98) | 99.98% (99.88-100) | 0.157 | 99.02% (98.27-99.69) | 0.099 |
| PPV | 70.93% (65.24-76.54) | 77.75% (72.34-83.03) | 0.004 | 68.11% (62.48-73.67) | 0.190 |
| Sens | 99.11% (97.65-100) | 100% (100-100) | 0.157 | 97.33% (94.98-99.18) | 0.102 |
| Spec | 93.53% (92.13-94.93) | 95.34% (94.03-96.5) | 0.005 | 92.34% (90.68-93.81) | 0.085 |

| Subgroup - no renal disease (eGFR ≥60 mL/min/1.73 m) | | |  |  |  |  |
| --- | --- | --- | --- | --- | --- | --- |
| n=1106 (128) | cMyC | | hs-cTnT | p* | hs-cTnI | p† |
| ﻿NPV | 99.61% (99.12-99.98) | 99.98% (99.87-100) | | 0.157 | 99.28% (98.6-99.85) | 0.305 |
| PPV | 72.97% (66.29-79.49) | 80.52% (74.33-86.49) | | 0.009 | 67.97% (61.35-74.49) | 0.064 |
| Sens | 98.73% (96.75-100) | 100% (100-100) | | 0.157 | 97.45% (94.84-99.39) | 0.317 |
| Spec | 95.15% (93.83-96.45) | 96.73% (95.49-97.8) | | 0.011 | 93.58% (91.85-95.02) | 0.025 |

| Subgroup - renal disease (eGFR <60 mL/min/1.73 m) | | | | | |
| --- | --- | --- | --- | --- | --- |
| n=211 (48) | cMyC | hs-cTnT | p* | hs-cTnI | p† |
| ﻿NPV | 98.57% (94.55-100) | 99.55% (97.59-100) | 1.000 | 93.75% (86.35-99.57) | 0.157 |
| PPV | 65.07% (54.14-75.77) | 70.77% (60.02-81.18) | 0.186 | 67.36% (56.53-77.93) | 0.508 |
| Sens | 100% (100-100) | 100% (100-100) | 1.000 | 97.05% (92.31-100) | 0.157 |
| Spec | 82.79% (76.22-89.04) | 86.33% (80.4-91.78) | 0.197 | 84.29% (78.57-89.58) | 0.564 |

Table S6 – All patients and subgroups as per presence or absence of renal dysfunction; NPV = Negative Predictive Value; PPV = Positive Predictive Value; Sens = Sensitivity (for rule-out); Spec = Specificity (for rule-in); * p value for comparison cMyC & hs-cTnT; † p value for comparison cMyC & hs-cTnI;

## cMyC pathway in male patients

| Subgroup - male patients | | | | | |  |
| --- | --- | --- | --- | --- | --- | --- |
| n=907 (125) | cMyC | hs-cTnT | p* | hs-cTnI | p† | |
| ﻿NPV | 99.67% (99.14-100) | 99.97% (99.82-100) | 0.317 | 98.78% (97.75-99.67) | 0.045 | |
| PPV | 74.4% (67.72-80.91) | 82.78% (76.72-88.6) | 0.004 | 70.83% (64.16-77.37) | 0.227 | |
| Sens | 99.42% (98.06-100) | 100% (100-100) | 0.317 | 96.98% (94.35-99.38) | 0.046 | |
| Spec | 94.36% (92.63-95.94) | 96.5% (95.11-97.79) | 0.005 | 92.98% (91.11-94.75) | 0.140 | |

Table S7 – Subgroup male patients

## cMyC pathway in female patients

| Subgroup - female patients | | | | | |  |
| --- | --- | --- | --- | --- | --- | --- |
| n=419 (53) | cMyC | hs-cTnT | p* | hs-cTnI | p† | |
| ﻿NPV | 99.32% (98.24-100) | 99.93% (99.64-100) | 0.317 | 99.31% (98.2-100) | 0.990 | |
| PPV | 63.58% (53.11-73.86) | 68.14% (57.85-78.17) | 0.258 | 62.21% (51.98-72.28) | 0.612 | |
| Sens | 98.29% (93.94-100) | 100% (100-100) | 0.317 | 98.22% (94.03-100) | 1.000 | |
| Spec | 91.9% (88.98-94.45) | 93.09% (90.44-95.46) | 0.346 | 91.17% (88.02-94.06) | 0.366 | |

Table S8 – Subgroup female patients; NPV = Negative Predictive Value; PPV = Positive Predictive Value; Sens = Sensitivity (for rule-out); Spec = Specificity (for rule-in); * p value for comparison cMyC & hs-cTnT; † p value for comparison cMyC & hs-cTnI

## cMyC pathway in patients with age ≤65y

| Subgroup - age ≤65 | | | | | | |
| --- | --- | --- | --- | --- | --- | --- |
| n=735 (70) | cMyC | hs-cTnT | p* | hs-cTnI | p† |  |
| ﻿NPV | 99.7% (99.22-100) | 99.97% (99.84-100) | 0.317 | 99.9% (99.62-100) | 0.317 |  |
| PPV | 74.21% (65.37-82.76) | 77.84% (69.25-86.08) | 0.326 | 71.63% (62.94-80.1) | 0.432 |  |
| Sens | 98.79% (95.95-100) | 100% (100-100) | 0.317 | 100% (100-100) | 0.317 |  |
| Spec | 96.27% (94.85-97.73) | 96.92% (95.52-98.18) | 0.317 | 95.51% (93.87-96.97) | 0.251 |  |

Table S9 – Subgroup patients aged ≤65 years;

| Subgroup - age >65 years | | | |  |  |  |
| --- | --- | --- | --- | --- | --- | --- |
| n=591 (108) | cMyC | hs-cTnT | p* | | hs-cTnI | p† |
| ﻿NPV | 99.12% (97.72-100) | 99.91% (99.53-100) | 0.317 | | 96.08% (93.1-98.73) | 0.024 |
| PPV | 68.75% (61.36-76.01) | 77.74% (70.8-84.45) | 0.004 | | 65.74% (58.42-72.96) | 0.284 |
| Sens | 99.28% (97.69-100) | 100% (100-100) | 0.317 | | 95.73% (92.2-98.59) | 0.025 |
| Spec | 89.55% (86.71-92.21) | 93.13% (90.65-95.39) | 0.006 | | 87.82% (84.8-91.01) | 0.194 |

Table S10 – Subgroup patients aged >65 years; NPV = Negative Predictive Value; PPV = Positive Predictive Value; Sens = Sensitivity (for rule-out); Spec = Specificity (for rule-in); * p value for comparison cMyC & hs-cTnT; † p value for comparison cMyC & hs-cTnI

## cMyC pathway in very early, early and late presenters

| \| Subgroup - very early presenters (≤2 hours of chest pain) \| \| \| \| \| \| \| --- \| --- \| --- \| --- \| --- \| --- \| \| n=341 (52) \| cMyC \| hs-cTnT \| p* \| hs-cTnI \| p† \| \| ﻿NPV \| 99.73% (98.98-100) \| 99.92% (99.56-100) \| 1.000 \| 99.22% (97.97-100) \| 0.317 \| \| PPV \| 75.69% (65.75-85.24) \| 80.05% (70.35-89.22) \| 0.239 \| 72.3% (62.07-82.18) \| 0.299 \| \| Sens \| 100% (100-100) \| 100% (100-100) \| 1.000 \| 98.43% (94.74-100) \| 0.317 \| \| Spec \| 93.86% (90.77-96.62) \| 95.26% (92.45-97.53) \| 0.157 \| 92.75% (89.72-95.6) \| 0.317 \|  \| Subgroup - early presenters (≤3 hours of chest pain) \| \| \| \| \| \| \| \| --- \| --- \| --- \| --- \| --- \| --- \| --- \| \| n=488 (63) \| cMyC \| hs-cTnT \| p* \| hs-cTnI \| p† \| \| \| ﻿NPV \| 99.81% (99.29-100) \| 99.94% (99.7-100) \| 1.000 \| 99.45% (98.57-100) \| \| 0.317 \| \| \| PPV \| 73.3% (64.02-82.28) \| 73.37% (64.04-82.39) \| 0.926 \| 66.15% (56.67-75.43) \| \| 0.033 \| \| \| Sens \| 100% (100-100) \| 100% (100-100) \| 1.000 \| 98.71% (95.77-100) \| \| 0.317 \| \| \| Spec \| 94.37% (92.05-96.41) \| 94.39% (92.23-96.57) \| 1.000 \| 92.16% (89.5-94.74) \| \|  \| \|  \| Subgroup - late presenters (>3 hours of chest pain) \| \| \| \| \| --- \| --- \| --- \| --- \| \| n=838 (115) \| cMyC \| hs-cTnT \| p* \| hs-cTnI \| p† \| \| ﻿NPV \| 99.38% (98.62-99.96) \| 99.96% (99.8-100) \| 0.157 \| 98.61% (97.44-99.62) \| 0.169 \| \| PPV \| 69.5% (62.31-76.54) \| 80.44% (73.9-86.73) \| 0.001 \| 69.12% (62.15-75.96) \| 0.890 \| \| Sens \| 98.69% (96.53-100) \| 100% (100-100) \| 0.157 \| 96.68% (93.51-99.28) \| 0.180 \| \| Spec \| 93.06% (91.09-94.96) \| 95.94% (94.35-97.34) \| 0.001 \| 92.46% (90.37-94.41) \| 0.527 \|   Table S11 – Subgroups stratified as per time since chest pain onset; NPV = Negative Predictive Value; PPV = Positive Predictive Value; Sens = Sensitivity (for rule-out); Spec = Specificity (for rule-in); * p value for comparison cMyC & hs-cTnT; † p value for comparison cMyC & hs-cTnI |
| --- | --- | --- | --- | --- | --- | --- | --- | --- | --- | --- | --- | --- | --- | --- | --- | --- | --- | --- | --- | --- | --- | --- | --- | --- | --- | --- | --- | --- | --- | --- | --- | --- | --- | --- | --- | --- | --- | --- | --- | --- | --- | --- | --- | --- | --- | --- | --- | --- | --- | --- | --- | --- | --- | --- | --- | --- | --- | --- | --- | --- | --- | --- | --- | --- | --- | --- | --- | --- | --- | --- | --- | --- | --- | --- | --- | --- | --- | --- | --- | --- | --- | --- | --- | --- | --- | --- | --- | --- | --- | --- | --- | --- | --- | --- | --- | --- | --- | --- | --- | --- | --- | --- | --- | --- | --- | --- | --- | --- | --- | --- | --- | --- | --- | --- | --- | --- |

| n=1326 (178 NSTEMI) | cMyC 0/1h-algorithm | hs-cTnT 0/1h-algorithm | p* |
| --- | --- | --- | --- |
| Prevalence of NSTEMI | 17% (same cohort) | |  |
| NPV | 99.63% (99.17-99.98) | 99.93% (99.75-100) | 0.157 |
| Sensitivity | 99.13% (97.73-100) | 100% (100-100) | 0.157 |
| PPV | 70.93% (65.24-76.54) | 77.61% (72.18-82.09) | 0.004 |
| Specificity | 93.51% (92.03-95) | 95.35% (94.19-96.6) | 0.005 |
| LR+ | 11.93 (9.53-15.37) | 16.99 (13.11-22.96) |  |
| LR- | 0.01 (0-0.02) | 0 (0-0.01) |  |
| Proportion ruled-out |  |  |  |
| Based on 0-hour sample | 33.8% | 9.5% | <0.001 |
| Based on 0/1-hour samples | 50.7& | 57.4% | 0.001 |
| Proportion ruled-in |  |  |  |
| Based on 0-hour sample | 12.7% | 11.2% | 0.281 |
| Based on 0/1-hour samples | 18.5% | 17.3 | 0.447 |
| Overall efficacy |  |  |  |
| Based on 0-hour sample | 46.5% | 20.7% | <0.001 |
| Based on 0/1-hour samples | 69.2% | 74.6% | 0.002 |
| Prevalence of NSTEMI in observational group | 12.2% | 14.3% | 0.472 |

Table S12 – Direct comparison of the performance of the cMyC 0/1h-algorithm to the established ESC hs-cTnT 0/1h-algorithm in all patients; NPV = Negative Predictive Value; PPV = Positive Predictive Value; LR- = Negative Likelihood Ratio; LR+ = Positive Likelihood Ratio; NSTEMI = Non-ST elevation Myocardial Infarction; * p values for comparison cMyC to hs-cTnT;

## Dual-marker strategy – Algorithm using ESC hs-cTnT 0/1h + cMyC 0h compared to ESC hs-cTnT 0/1h algorithm alone

| N=663 (114 NSTEMI) | cMyC at 0h + hs-cTnT 0/1h algorithm | hs-cTnT 0/1h algorithm | | p* |
| --- | --- | --- | --- | --- |
| Prevalence of NSTEMI | 17% (same cohort) | |  | |
| ﻿NPV | 99.87% (99.52-100) | 99.87% (99.51-100) | | 1.000 |
| PPV | 75.6% (68.03-82.94) | 78.88% (71.4-86.08) | | 0.038 |
| Sens | 100% (100-100) | 100% (100-100) | | 1.000 |
| Spec | 94.56% (92.6-96.46) | 95.61% (93.74-97.21) | | 0.014 |
| ﻿LR+ | 15.09 (10.78-22.43) | 18.26 (12.56-28.66) | |  |
| LR- | 0 (0-0.02) | 0 (0-0.03) | |  |
| Proportion ruled-out |  |  | |  |
| Based on 0-hour sample | ﻿38.5% | 10.9% | | 0.000 |
| Based on 0/1-hour samples | ﻿60.0% | 58.8% | | 0.695 |
| Proportion ruled-in |  |  | |  |
| Based on 0-hour sample | ﻿13.3% | 10.3% | | 0.105 |
| Based on 0/1-hour samples | ﻿18.7% | 17.4% | | 0.568 |
| Overall efficacy |  |  | |  |
| Based on 0-hour sample | ﻿51.7% | 21.1% | | 0.000 |
| Based on 0/1-hour samples | ﻿78.7% | 76.2% | | 0.293 |
| Prevalence of NSTEMI in observational group | ﻿14.2% | 14.6% | | 1.000 |

Table S13 – Direct comparison of the performance of a modified ESC hs-cTnT 0/1h-algorithm with the addition of the cMyC triage booster; NPV = Negative Predictive Value; PPV = Positive Predictive Value; LR- = Negative Likelihood Ratio; LR+ = Positive Likelihood Ratio; NSTEMI = Non-ST elevation Myocardial Infarction; * p values for comparison modified ESC pathway to the established ESC hs-cTnT 0/1h-algorithm

## Dual-marker strategy – Algorithm using ESC hs-cTnI 0/1h + cMyC 0h compared to ESC hs-cTnI 0/1h algorithm alone

| N=663 (114 NSTEMI) | cMyC at 0h + hs-cTnI 0/1h algorithm | | hs-cTnI 0/1h algorithm | | | p* | |
| --- | --- | --- | --- | --- | --- | --- | --- |
| Prevalence of NSTEMI | 17% (same cohort) | | |  | | | |
| ﻿NPV | 99.58% (98.9-100) | 99.56% (98.85-100) | | | 0.334 | |  |
| PPV | 71.92% (64.16-79.49) | 72.27% (64.47-79.86) | | | 0.728 | |  |
| Sens | 99.11% (96.81-100) | 99.12% (97.03-100) | | | 1.000 | |  |
| Spec | 93.45% (91.4-95.38) | 93.59% (91.43-95.45) | | | 0.564 | |  |
| ﻿LR+ | 12.44 (9.12-17.75) | 12.66 (9.26-18.27) | | |  | |  |
| LR- | 0.01 (0-0.03) | 0.01 (0-0.03) | | |  | |  |
| Proportion ruled-out |  |  | | |  | |  |
| Based on 0-hour sample | 41.9% | 18.9% | | | <0.001 | |  |
| Based on 0/1-hour samples | 53.5% | 51.4% | | | 0.475 | |  |
| Proportion ruled-in |  |  | | |  | |  |
| Based on 0-hour sample | 13.9% | 11% | | | 0.134 | |  |
| Based on 0/1-hour samples | 19.5% | 19.2% | | | 0.944 | |  |
| Overall efficacy |  |  | | |  | |  |
| Based on 0-hour sample | 55.8% | 29.9% | | | <0.001 | |  |
| Based on 0/1-hour samples | 73% | 70.6% | | | 0.360 | |  |
| Prevalence of NSTEMI in observational group | 11.2% | 10.8% | | | 1.000 | |  |

Table S14 – Direct comparison of the performance of a modified ESC hs-cTnI 0/1h-algorithm with the addition of the cMyC triage booster; NPV = Negative Predictive Value; PPV = Positive Predictive Value; LR- = Negative Likelihood Ratio; LR+ = Positive Likelihood Ratio; NSTEMI = Non-ST elevation Myocardial Infarction; * p values for comparison modified ESC pathway to the established ESC hs-cTnI 0/1h-algorithm

| No AMI | hs-cTnT |  | AMI | hs-cTnT |  | |  |
| --- | --- | --- | --- | --- | --- | --- | --- |
| cMyC | ≥99^th^ centile | <99^th^ centile |  | ≥99^th^ centile | | <99^th^ centile | |
| >99^th^ centile | 70 | 7 |  | 153 | | 1 | |
| <99^th^ centile | 190 | 833 |  | 50 | | 22 | |

| No AMI | hs-cTnI |  | AMI | hs-cTnI |  |
| --- | --- | --- | --- | --- | --- |
| cMyC | ≥99^th^ centile | <99^th^ centile |  | ≥99^th^ centile | <99^th^ centile |
| >99^th^ centile | 56 | 21 |  | 139 | 15 |
| <99^th^ centile | 31 | 992 |  | 15 | 57 |

| No AMI | hs-cTnI |  | AMI | hs-cTnI |  |
| --- | --- | --- | --- | --- | --- |
| hs-cTnT | ≥99^th^ centile | <99^th^ centile |  | ≥99^th^ centile | <99^th^ centile |
| >99^th^ centile | 77 | 183 |  | 152 | 51 |
| <99^th^ centile | 10 | 830 |  | 2 | 21 |

Table S15 – 2x2 tables to assess for concordance and discordance against the assay-specific 99^th^ centiles: cMyC 87 ng/L, hs-cTnT 14 ng/L, hs-cTnI 26 ng/L

## Supplemental Figures

### Study recruitment


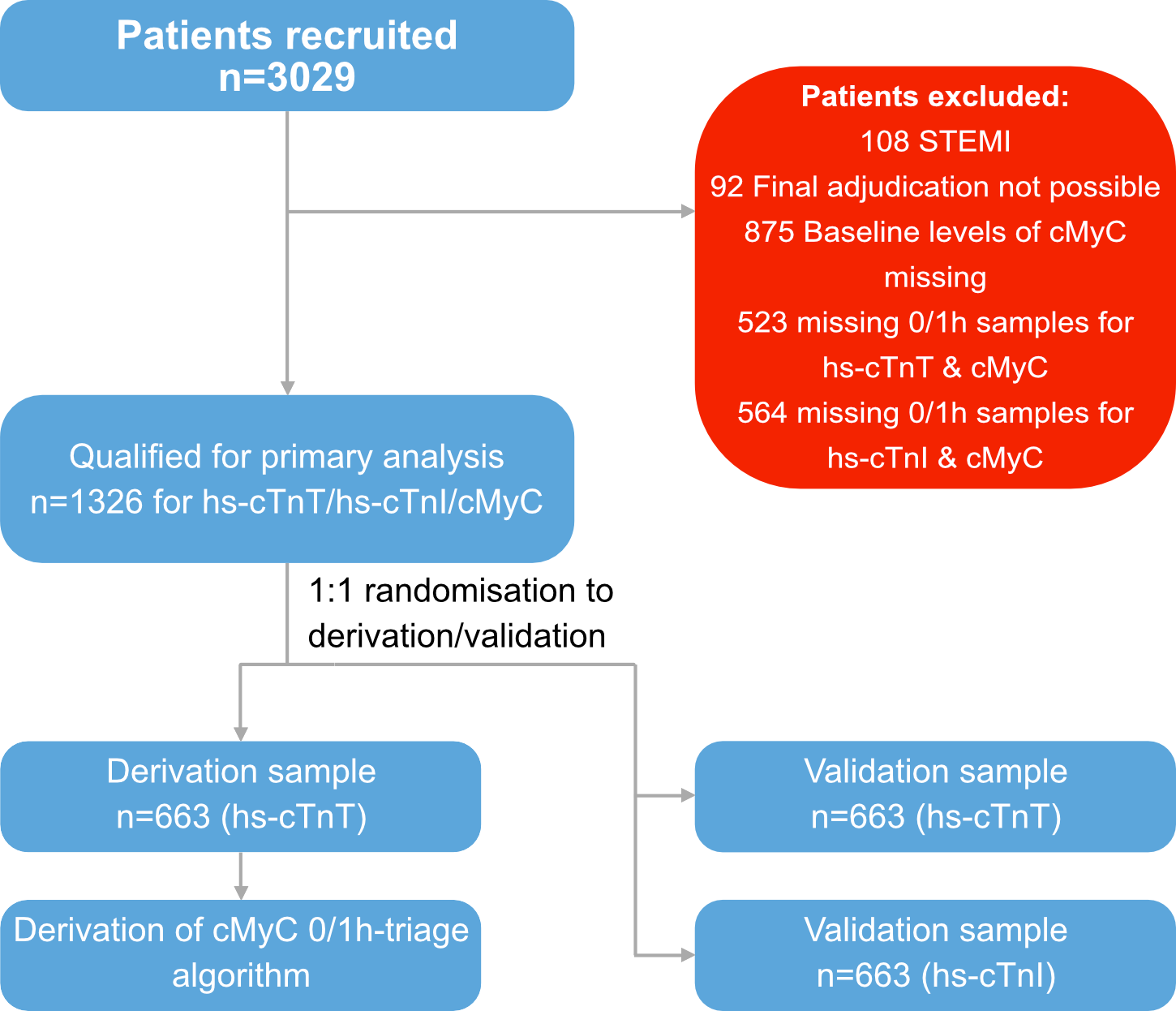


Figure S1 – Flow chart describing the study recruitment and cohort split into derivation & validation


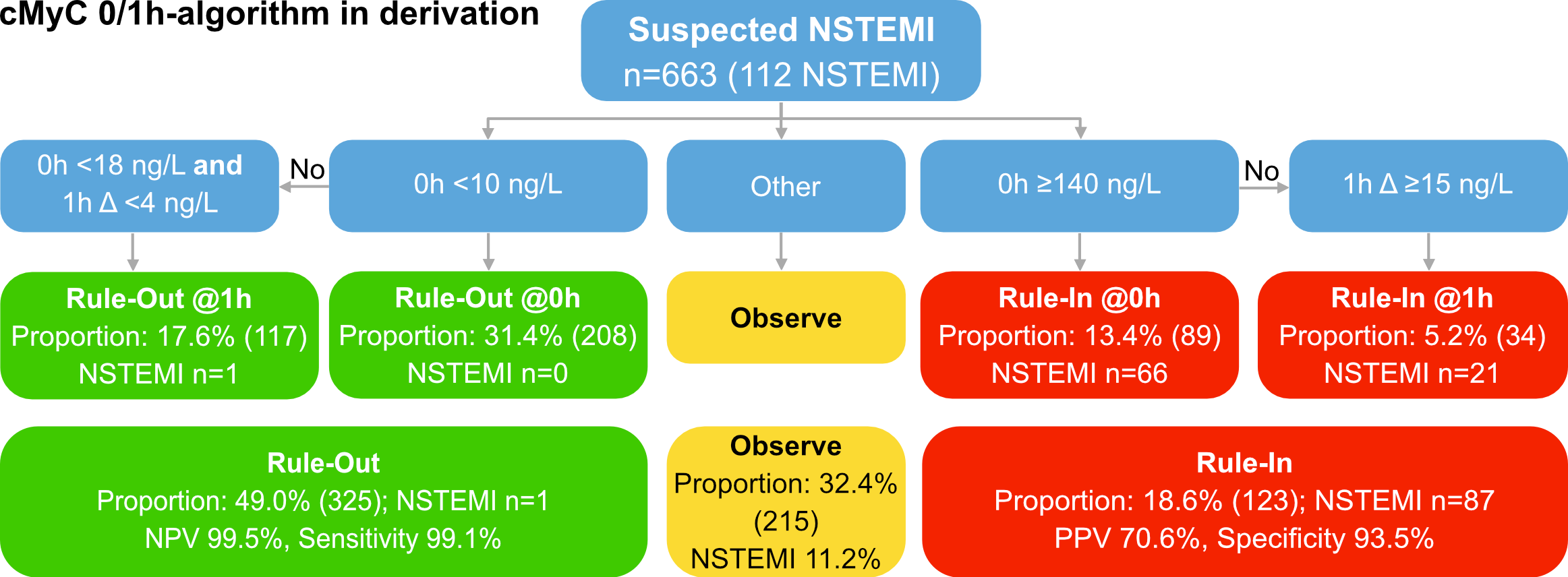


Figure S2 – cMyC 0/1h-algorithm: modelled on the ESC 0/1h-hs-cTnT/I-algorithms(14). Thresholds and performance metrics obtained in the derivation cohort; the cMyC 0/1h-algorithm can be used irrespective of time interval from symptom onset to first blood draw.


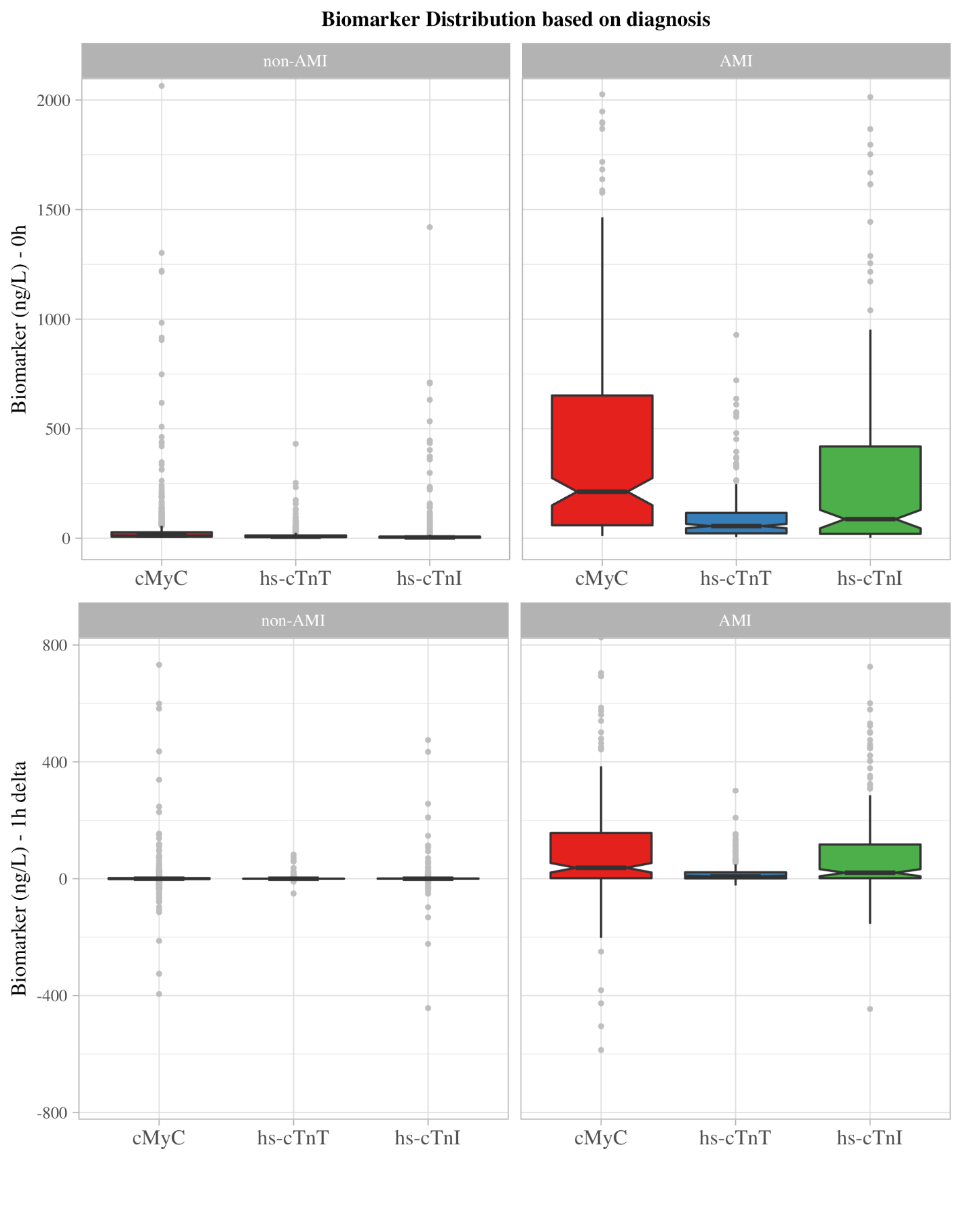


Figure S3 – Biomarker distribution based on final diagnosis of NSTEMI vs Non-AMI group; x-axis biomarkers cMyC, hs-cTnT and hs-cTnI; y-axis depicts median biomarker concentration in ng/L. Lower and upper hinges correspond to the first and third quartiles (the 25th and 75th percentiles); upper & lower whiskers extend from the hinge to the largest/smallest value no further than 1.5 * IQR from the hinge. Data beyond the end of the whiskers are ‘outlying’ points and are plotted individually; the notches extend 1.58 * IQR / sqrt(n), providing an approximation to the 95% confidence interval for comparing medians.

### Distribution of biomarker concentrations for 0h, 1h samples and delta-change – ratio biomarker/99^th^ centile and LOD


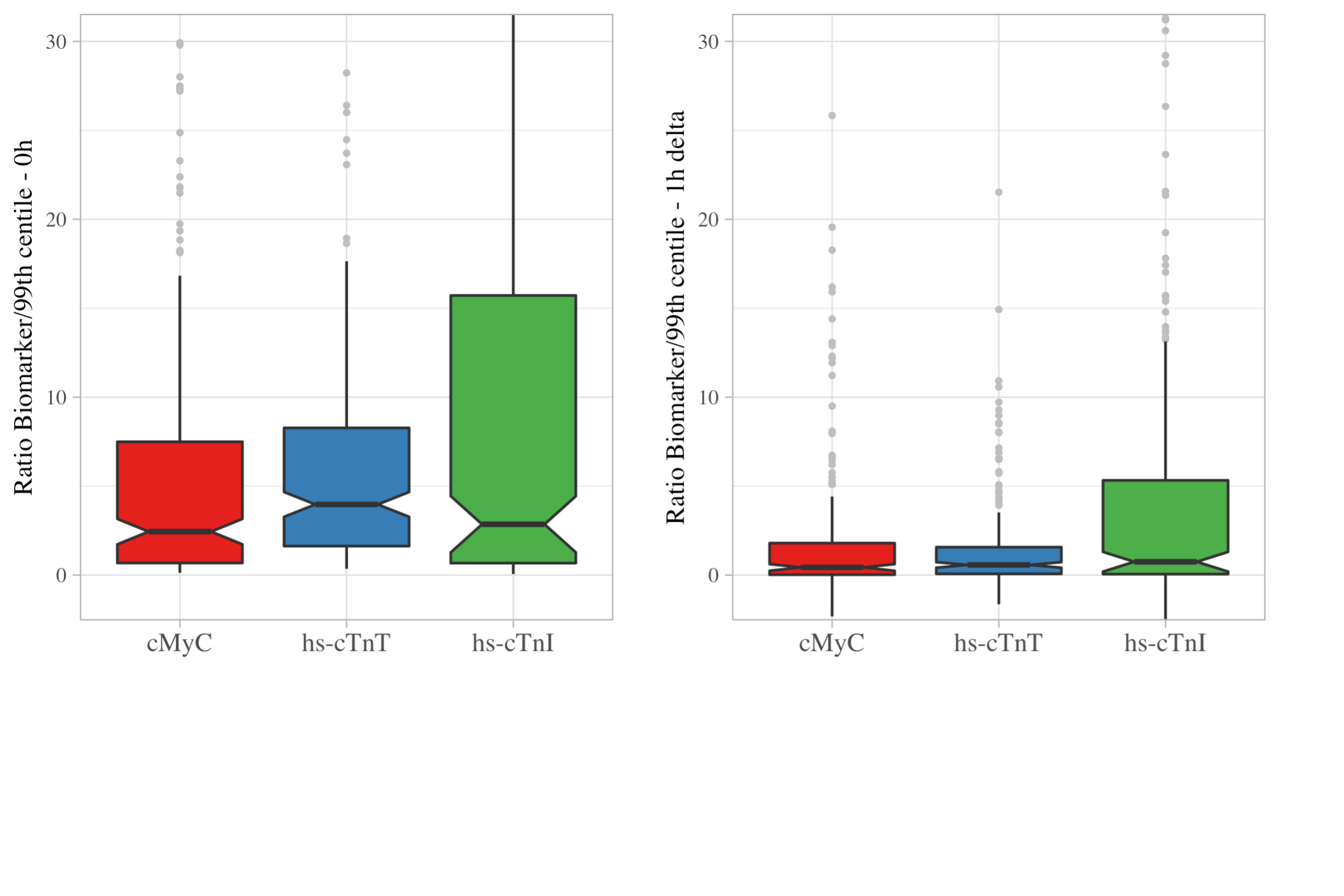


Figure S4 – Biomarker distribution based on final diagnosis of NSTEMI; x-axis biomarkers cMyC, hs-cTnT and hs-cTnI; y-axis depicts median ratio of biomarker/99^th^ centile (87 ng/L for cMyC, 14 ng/L for hs-cTnT, sex-specific cut-offs at 34 ng/L (male) and 16 ng/L (female) for hs-cTnI). Lower and upper hinges correspond to the first and third quartiles (the 25th and 75th percentiles); upper & lower whiskers extend from the hinge to the largest/smallest value no further than 1.5 * IQR from the hinge. Data beyond the end of the whiskers are ‘outlying’ points and are plotted individually; the notches extend 1.58 * IQR / sqrt(n), providing an approximation to the 95% confidence interval for comparing medians.

## Forrest plot


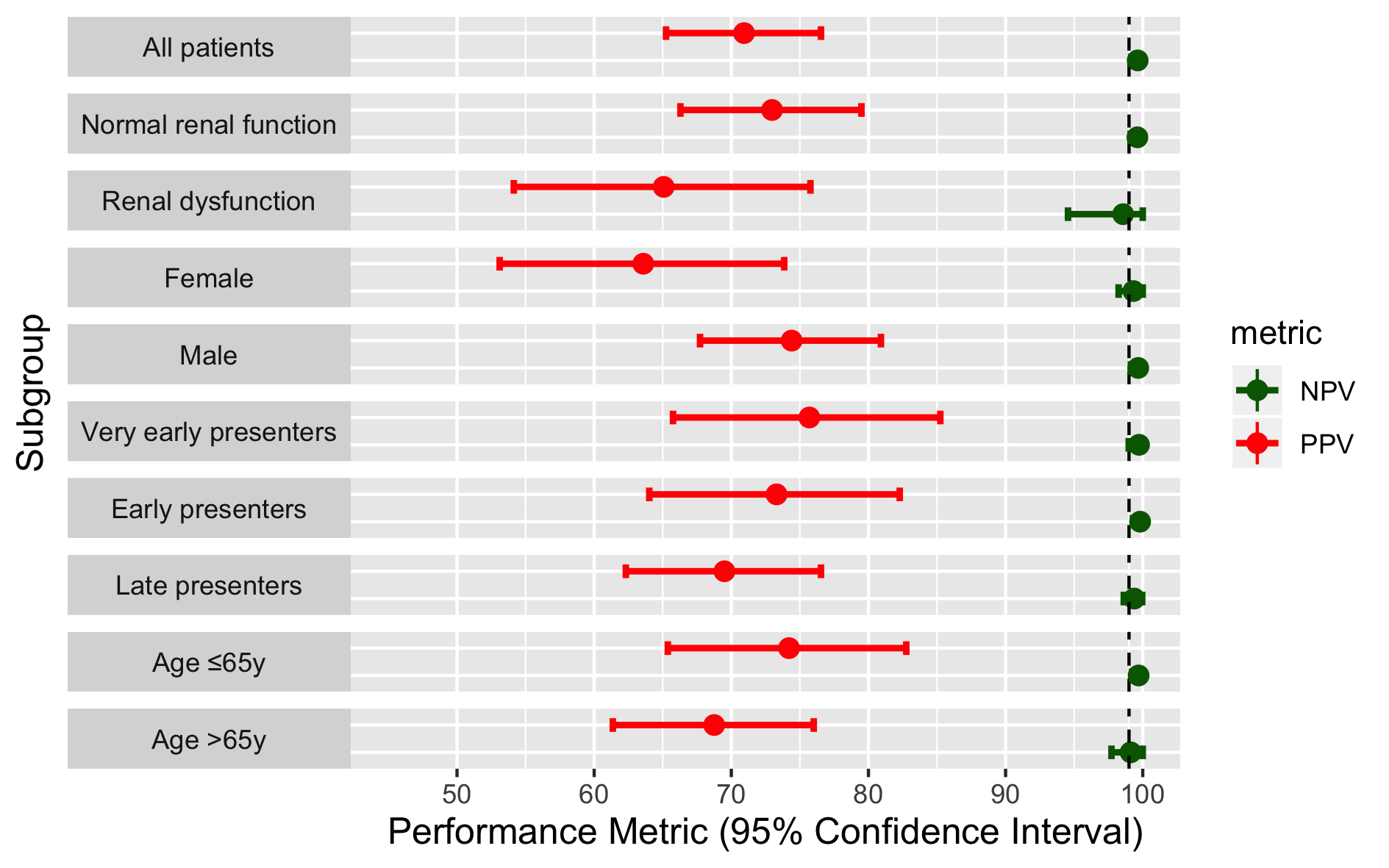


Figure S5 – Performance analysis for the cMyC 0/1h-algorithm in multiple, pre-defined subgroups; vertical black line indicates performance metric of 99% (pre-defined target for NPV); ‘Normal renal function’ = eGFR ≥60 mL/min/1.73m; ‘Renal dysfunction’ = eGFR <60 mL/min/1.73m; ‘Very early presenters’ = chest pain ≤2 hours; ‘Early presenters’ = chest pain ≤3 hours; ‘Late presenters’ = chest pain >3 hours; p>0.05 (for interaction) for comparison between all subgroups


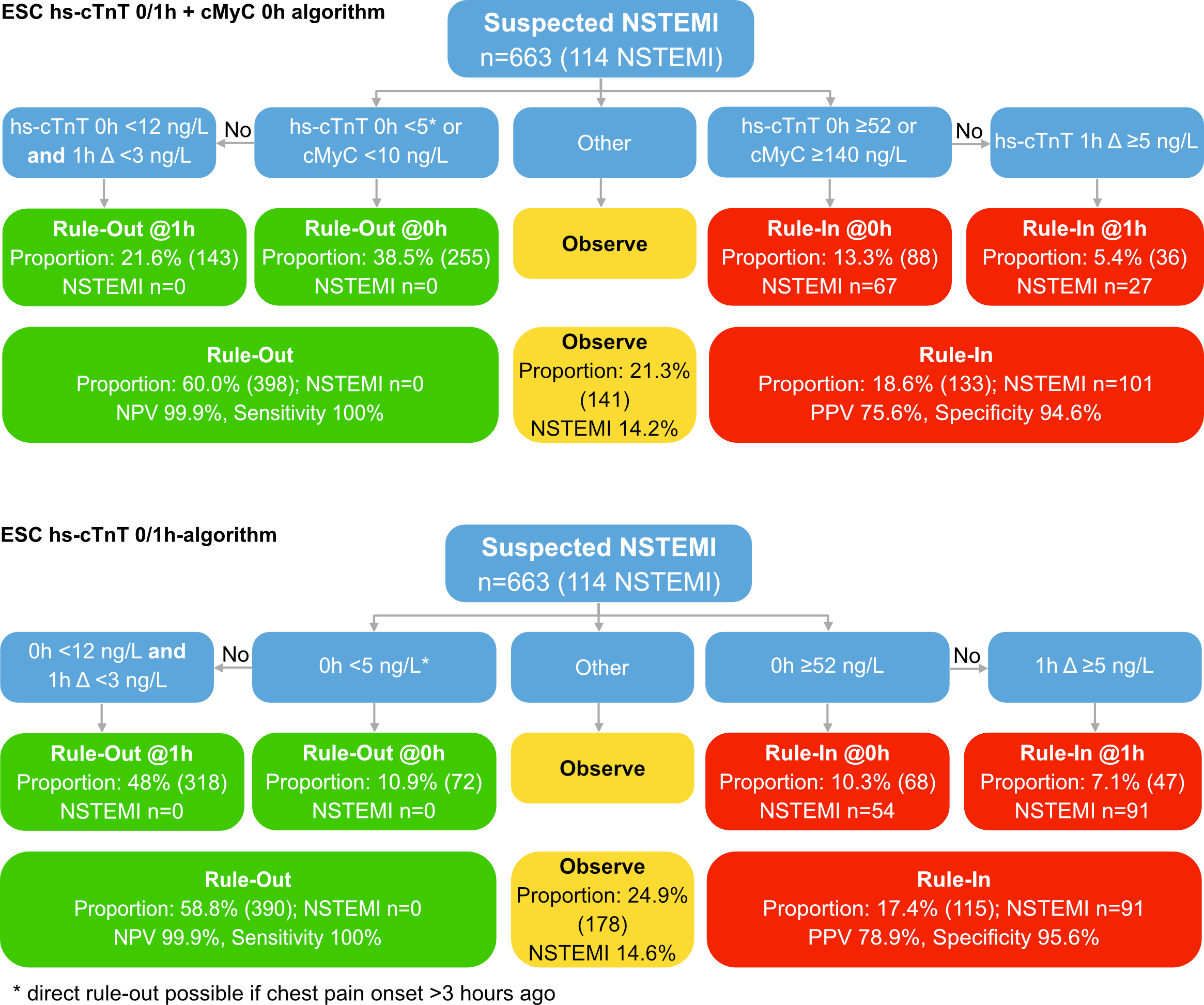


Figure S6 – Direct comparison of patient distribution in the validation cohort between the modified ESC hs-cTnT 0/1h-algorithm with the addition of the cMyC triage booster, and the ESC hs-cTnT 0/1h-algorithm


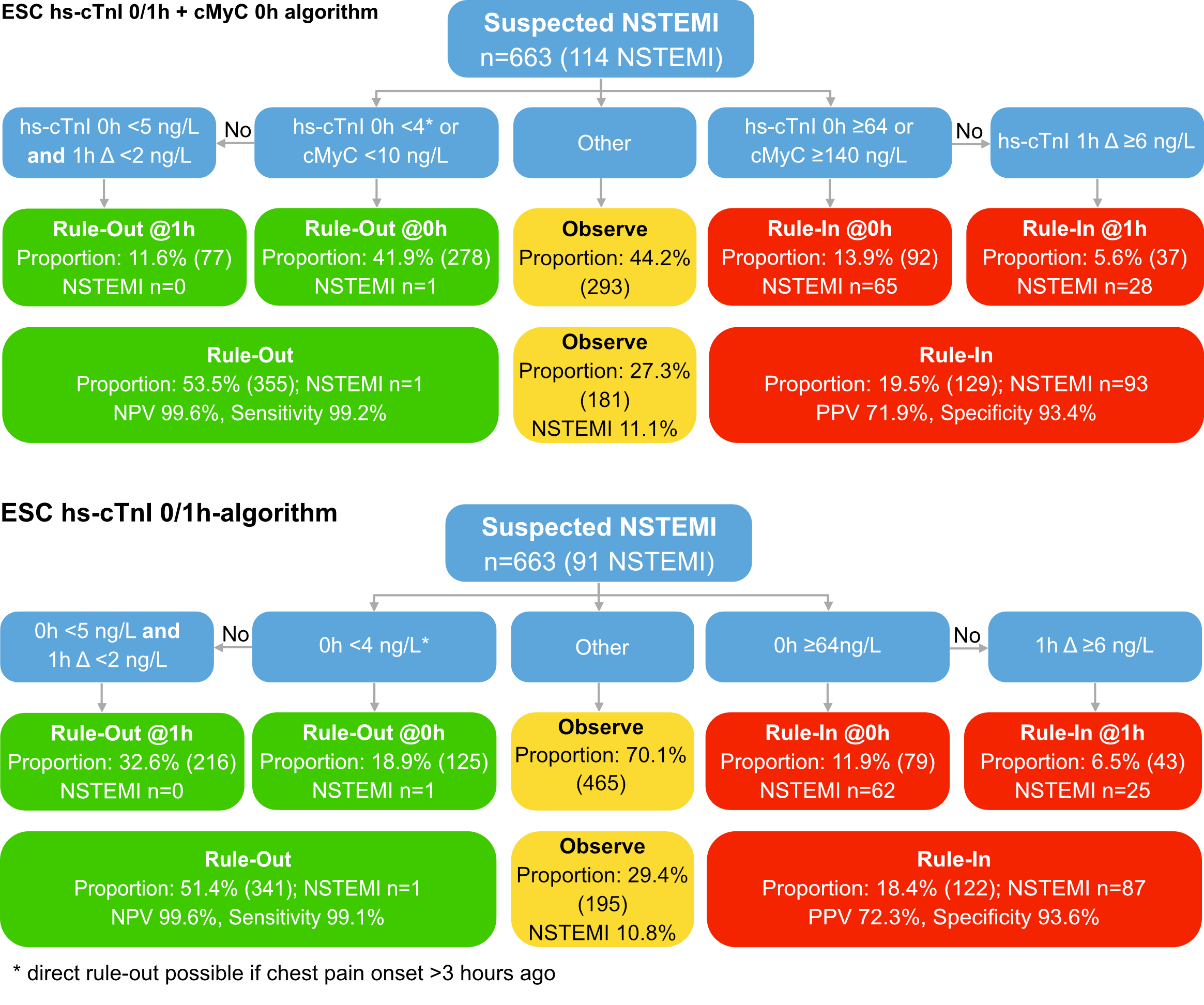


Figure S7 – Direct comparison of patient distribution in the validation cohort between the modified ESC hs-cTnI 0/1h-algorithm with the addition of the cMyC triage booster, and the ESC hs-cTnI 0/1h-algorithm


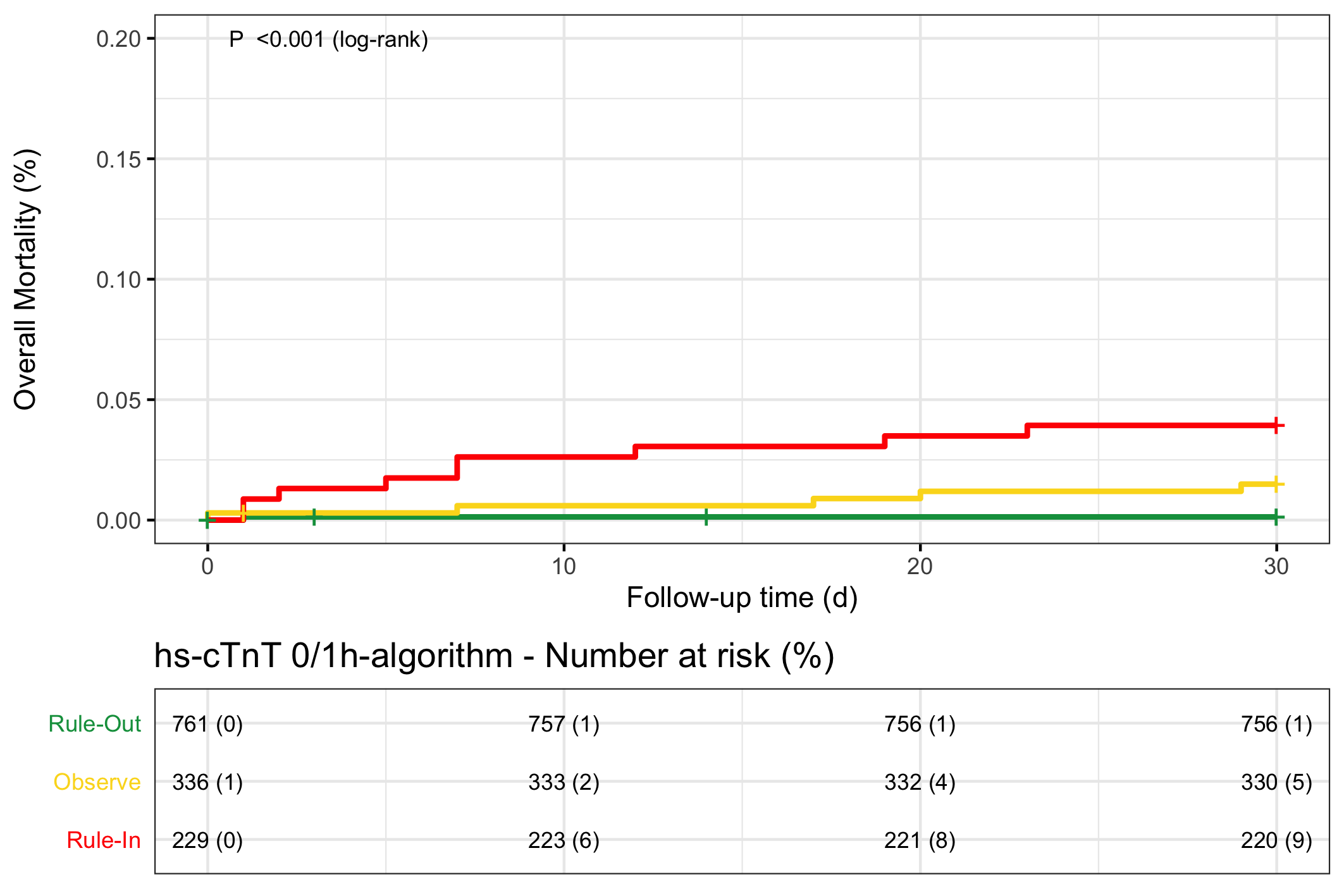

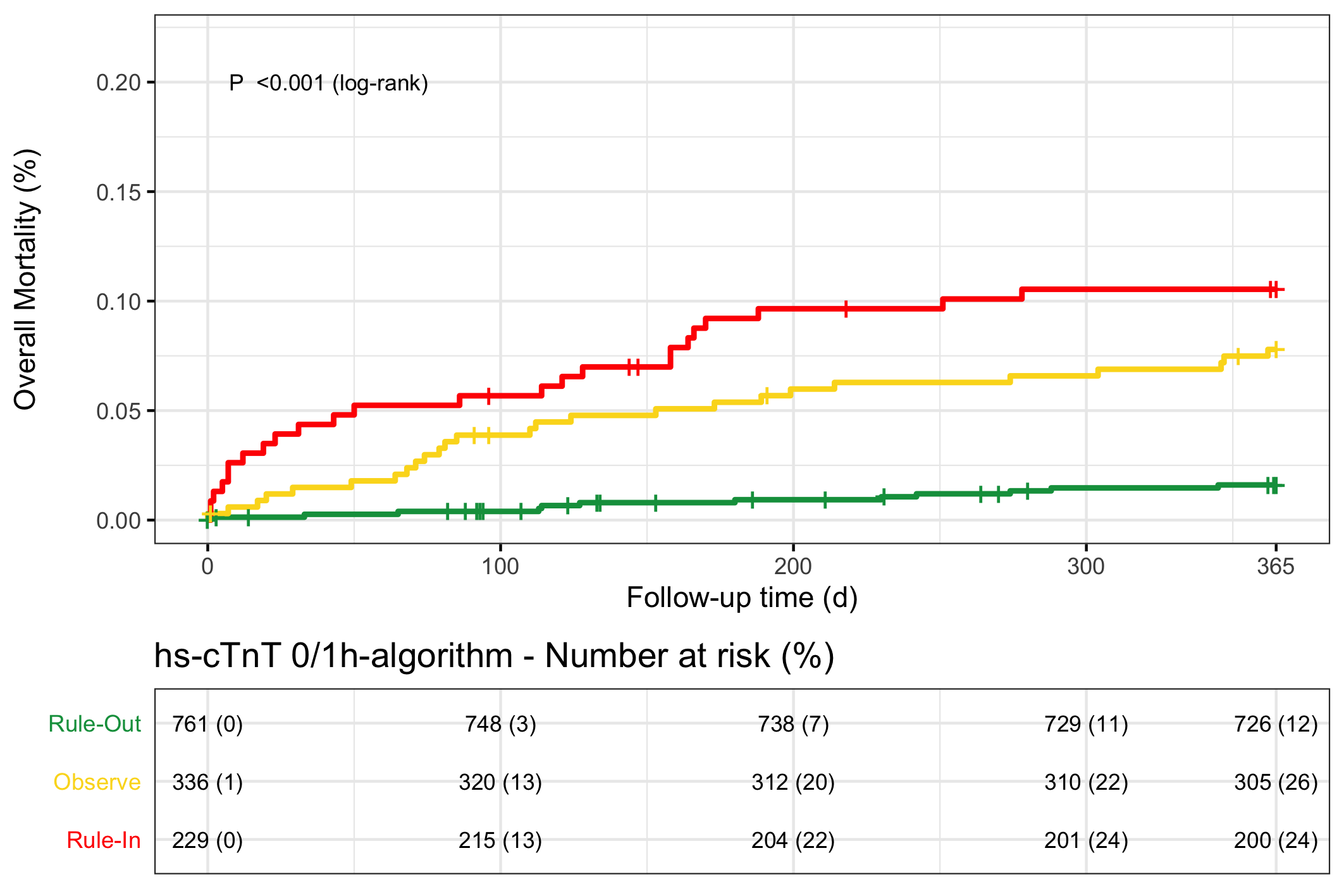


Figure S8 - Cumulative event (mortality) plot for the ESC hs-cTnT 0/1h-algorithm, with statistical comparison of event curves at 30d (top) and 365d (bottom) with log-rank tests; table displays number-at-risk and absolute number of events.


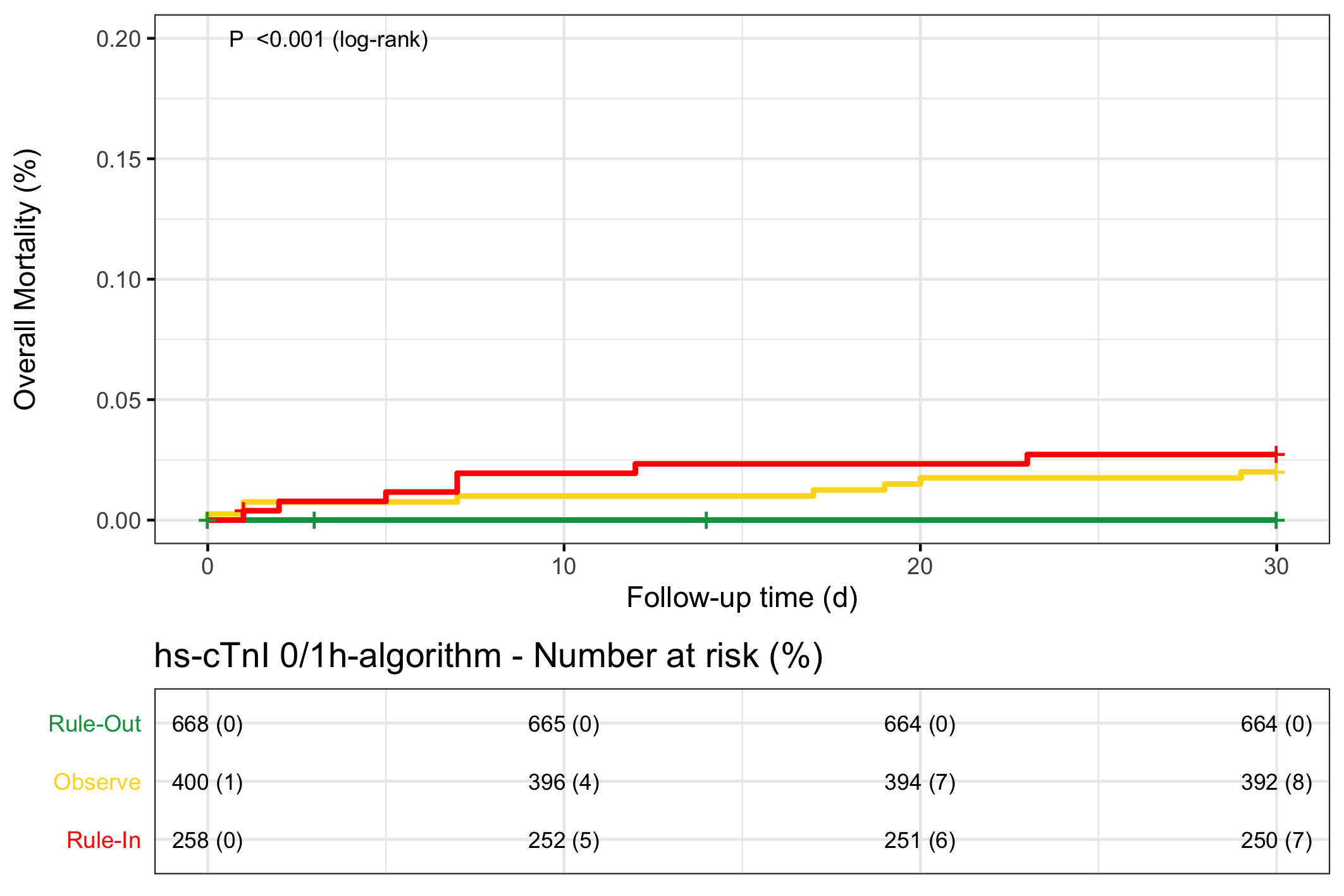


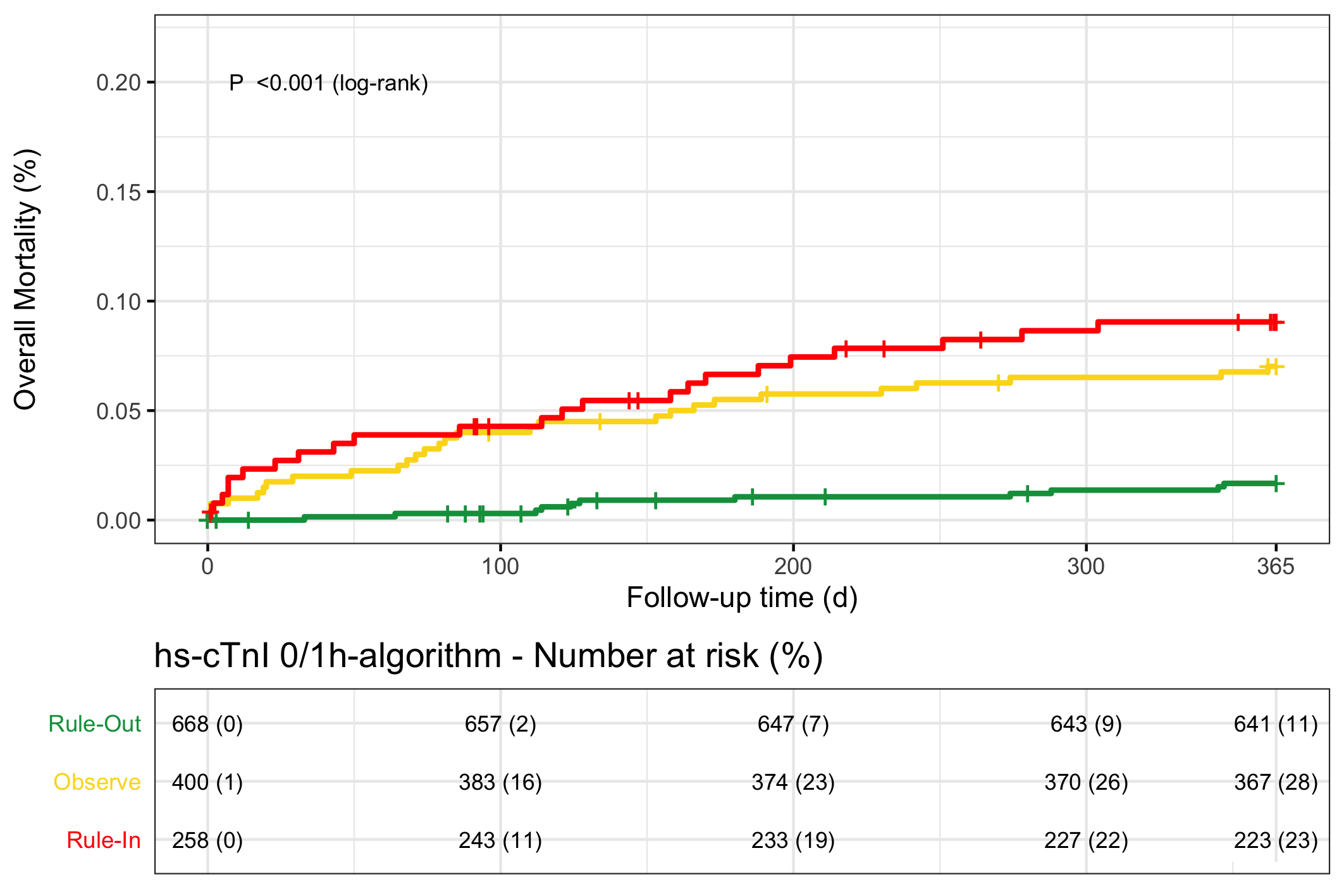


Figure S9 - Cumulative event (mortality) plot for the ESC hs-cTnI 0/1h-algorithm, with statistical comparison of event curves at 30d (top) and 365d (bottom) with log-rank tests; table displays number-at-risk and absolute number of events.

## References

1. Wu AHB, Lu QA, Todd J, Moecks J, Wians F. Short- and Long-Term Biological Variation in Cardiac Troponin I Measured with a High-Sensitivity Assay: Implications for Clinical Practice. Clinical Chemistry 2008;55:52–58.

2. Vasile VC, Saenger AK, Kroning JM, Jaffe AS. Biological and analytical variability of a novel high-sensitivity cardiac troponin T assay. Clinical Chemistry 2010;56:1086–1090.

3. Reichlin T, Irfan A, Twerenbold R, et al. Utility of Absolute and Relative Changes in Cardiac Troponin Concentrations in the Early Diagnosis of Acute Myocardial Infarction. Circulation 2011;124:136–145.

4. Hammarsten O, Fu MLX, Sigurjonsdottir R, et al. Troponin T Percentiles from a Random Population Sample, Emergency Room Patients and Patients with Myocardial Infarction. Clinical Chemistry 2012;58:628–637.

5. Marjot J, Liebetrau C, Goodson RJ, et al. The development and application of a high-sensitivity immunoassay for cardiac myosin-binding protein C. Translational research : the journal of laboratory and clinical medicine 2016;170:17–25.

6. Giannitsis E, Kurz K, Hallermayer K, Jarausch J, Jaffe AS, Katus HA. Analytical validation of a high-sensitivity cardiac troponin T assay. Clinical Chemistry 2010;56:254–261.

7. Wildi K, Twerenbold R, Jaeger C, et al. Clinical impact of the 2010-2012 low-end shift of high-sensitivity cardiac troponin T. European Heart Journal: Acute Cardiovascular Care 2016;5:399–408.

8. Koerbin G, Tate J, Potter JM, Cavanaugh J, Glasgow N, Hickman PE. Characterisation of a highly sensitive troponin I assay and its application to a cardio-healthy population. Clinical Chemistry and Laboratory Medicine 2012;50:1–8.

9. Kaier TE, Twerenbold R, Puelacher C, et al. Direct Comparison of Cardiac Myosin-Binding Protein C With Cardiac Troponins for the Early Diagnosis of Acute Myocardial Infarction. Circulation 2017;136:1495–1508.

10. NHS Institute for Innovation and Improvement. Reducing Length of Stay. Available at: http://webarchive.nationalarchives.gov.uk/20121108095055/http://www.institute.nhs.uk/quality_and_service_improvement_tools/quality_and_service_improvement_tools/length_of_stay.html. Accessed January 17, 2022.

11. UK Government. NHS Hospital Stay (2016). Available at: https://data.gov.uk/data-request/nhs-hospital-stay. Accessed January 17, 2022.

12. Goodacre SW. A prospective, observational study of a chest pain observation unit in a British hospital. Emergency Medicine Journal 2002;19:117–121.

13. Kaier TE, test. The use of a point-of-care device for the measurement of a novel biomarker in comparison to in-house diagnostic services in a central London teaching hospital – economic, legal and medical implications. Thesis, Middlesex University. 2017.

14. Collet J-P, Thiele H, Barbato E, et al. 2020 ESC Guidelines for the management of acute coronary syndromes in patients presenting without persistent ST-segment elevation. European Heart Journal 2020:ehaa575.
